# Manipulable Object and Human Contact: Preference and Modulation of Emotional States in Weaned Pigs

**DOI:** 10.3389/fvets.2020.577433

**Published:** 2020-11-27

**Authors:** Avelyne S. Villain, Mathilde Lanthony, Carole Guérin, Céline Tallet

**Affiliations:** PEGASE, INRAE, Institut Agro, Saint-Gilles, France

**Keywords:** enrichment, welfare, emotional reactivity, human-animal relationship, behavior, acoustic communication

## Abstract

Enriching the life of farm animals is a legal obligation in intensive farming conditions in the European Union, though not worldwide. In pigs, manipulable materials are mandatory when no bedding is available. Like manipulable objects, positive human interactions might also be considered as enrichment, as they provide the animals with opportunities to interact, increase their activity and lead to positive emotional states. In this study, we investigated how weaned pigs perceived an inanimate manipulable object and a familiar human. After a similar (in length, frequency, and procedure) familiarization to both stimuli, 24 weaned pigs were tested for a potential preference for one of the stimuli and submitted to isolation/reunion tests to evaluate the emotional value of the stimuli. We hypothesized that being reunited with a stimulus would attenuate the stress of social isolation and promote a positive state, especially if the stimulus had a positive emotional value for pigs. Although our behavioral data showed no evidence that pigs spent more time close to, or in contact with, one of the stimuli during a choice test, pigs more often approached the human and were observed lying down only near the human. Using behavioral and bioacoustic data from isolation/reunion tests, we showed that a reunion with the human decreased the time spent in an attentive state and mobility of pigs to a greater extent than a reunion with the object, or isolation. Vocalizations differed between reunions with the object and the human, and were different from those during isolation. The human and object presence led to higher frequency range and more noisy grunts, but only the human led to the production of positive shorter grunts, usually associated with positive situations. In conclusion, pigs seemed to be in a more positive emotional state, or be reassured, in the presence of a familiar human compared to the object after a short period of social isolation. This confirms the potential need for positive pseudo-social interactions with a human to enrich the pigs' environment, at least in or after potentially stressful situations.

## Introduction

An intensive production system for animal products sometimes implies high densities of farm animals and can lead to deleterious behaviors and decrease their physical or mental health, i.e., their welfare.

Animal welfare covers, among other things, the importance of the animal's ability to control its mental and physiological stability in different environmental conditions (1). Improving animal welfare involves both reducing negatively perceived contexts as well as increasing positively perceived contexts and species-specific behaviors (2, 3). The pressure from citizens, consumers, and animal welfare organizations regarding animal rights has been growing, leading to changes in the legislation. For example, the provision of manipulable materials to pigs of all ages has been mandatory in the European Union since January 2013 (4), using materials named as “environmental enrichments.” Environmental enrichments are defined as materials which can improve the biological functioning of captive animals (5) and should stimulate their species-typical sensory systems, cognitive capacities and behaviors (6). For instance, enrichments materials for pigs should be edible, chewable, investigable, and manipulable [(7) reviewed in (8)]. Moreover, enrichment materials should be provided in such a way that they offer sustainable attraction for pigs, should be accessible for oral manipulation, and provided in sufficient amount (5, 8). Enrichment effects are generally tested using behavioral and physiological paradigms (9) and are classified as optimal (if they meet all of the above-mentioned criteria), suboptimal (if meet most of the criteria but should be combined with other enrichment materials) or marginal [if they do not fulfill the animals' needs and should only be used with other enrichment materials (8)].

In the particular case of pigs, abnormal patterns of behavior (stereotypies, belly nosing, ear, and tail biting) may arise at several stages of their development if they are devoid of any enrichment (10). Enrichments have the potential to reduce these abnormal behaviors and increase positive behaviors like play (11, 12). Although straw bedding is one optimal enrichment according to literature [reviewed in (8)], it is laborious and costly to implement for farms using fully slatted floors. Thus, other manipulable materials in the form of ropes, hanging balls, wood, pipes, or different commercial toys have been developed and are used in farms.

Besides these enrichment materials, one may wonder if enrichment can be provided by other stimuli in the environment of farm animals. As pigs are social animals, social enrichment is sometimes used for nursing piglets, by allowing different litters to interact. This enrichment enhances play and decreases aggression at weaning (13). Another relational partner of pigs is their caregiver. Human interactions seem to correspond well to the definition of enrichment, i.e., they provide occasions of contact with another animal (stimulating biological functioning), and stimulate all sensory systems of the animals. Humans, notably through their clothes and boots, are chewable, investigable, and manipulable. Many beneficial outcomes of positive human interactions have been shown. Farm animals may be tamed by humans providing regular additional positive contacts, leading to the expression of positive emotions (14). Humans may consequently be associated with positive outcomes as measured by a decrease of heart rate (15–17), higher heart rate variability and indicative ear postures [ears hanging (17)], as well as their EEG [lower EEG total power and a shift in spectral power distribution toward higher frequencies (18)]. Humans can also acquire reassuring properties in situations of social isolation (19, 20). They may even induce behavioral reactions similar to those toward social partners (21). Cognitive bias tests showed a positive judgment bias in piglets that had received gentle contacts with humans (22). As positive judgment bias is often used to qualify the emotional state, this indicates that regular positive human contacts may lead to improved welfare. In addition, pigs raised in a poor environment may develop more interest toward a familiar human than pigs raised in an enriched environment (23), leading the author of the study to hypothesize that a familiar human may be perceived as an enrichment. To our knowledge, no comparison exists of the effect of inanimate object enrichment and pseudo-social enrichment via human interactions. This would provide new insight into enrichment practices for welfare improvement in pig breeding.

In this study, we developed a paradigm to test the perception pigs may have toward two stimuli: an inanimate object that could be used as enrichment, and a familiar human. The aim of this study was thus to elucidate the specific value for enrichment that a familiar human may have compared to an inanimate object. After familiarizing the pigs with each of them, we first evaluated the potential preference for one of the stimuli and then evaluated the emotional value of the stimuli through isolation/reunion tests. We hypothesized that being reunited with a stimulus would attenuate the stress of social isolation and promote positive behaviors (attraction toward the stimulus, contacts with the stimulus, play behaviors), especially if the stimulus has a higher positive emotional value for pigs. We used behavioral and bioacoustic data known to be relevant in comparing emotional states of pigs (24–26). Additionally, we tested if the level of attraction toward the stimuli could predict vocal expression in the presence of each stimulus.

## Materials and Methods

### Ethical Note

The experiment was carried out at the experimental farm UEPR, in Saint-Gilles, France, under the authorization no. APAFIS#17071-2018101016045373_V3 from the French Ministry of Higher Education, Research and Innovation, received after evaluation by the regional ethics committee (Comité Rennais d'Ethique en Experimentation Animale), and conformed with the French and European legislation regarding experiments on animals.

### Animals and Experimental Conditions

Twenty-four healthy weaned female pigs (Landrace/Large white dams inseminated with Piétrain semen) were involved in total. Pigs were weaned at 28 days of age. Eight groups of three sister pigs from eight different sows were selected at weaning according to their weight (the weight was balanced between and within the groups, 9.05± 0.66 kg on average). Thus, only familiar pigs from the same genetic mother were put together in rearing pens. Groups were housed in the same rearing room, in 115 × 132 cm pens, with slatted flooring, visually isolated from each other by 1.5 m high plastic panels. Pigs were fed *ad libitum* and had continuous access to a water trough. Each pen was provided with a steel chain as enrichment [mandatory by the Council Directive 2008/120/EC 2008 but demonstrated as low quality enrichment (8)]. The pigs were involved in the experiment from 28 to 57 days of age.

For several phases of the experiment, we also used an experimental room. This was located in the same building as the rearing room, 15 m away, and was a 270 cm × 270 cm soundproof room. It was warmed by an electric heater. The entrance door was equipped with a hatch for the pigs. The transportation from the rearing room to the experimental room was done by the usual caretakers in closed trolleys. We used visually isolated trolleys to transport either the group of three pigs together (L120 × W80 × H80 cm), if they were brought to the experimental room all together (familiarization sessions with stimuli in the experimental room, see below), or one pig for a time (L80 × W50 × H80 cm), if the pigs were brought to the experimental room for a test (Choice test and Isolation/Reunion test, see below).

### Human and Object Familiarization

All the pigs were familiarized with two stimuli: an experimenter, subsequently referred to as “Human” (always the same person, a 1.65 m tall woman dressed in a blue overall) and a 5L-plastic can (L20 × W10 × H30 cm), filled with water, from which hung three pipe pieces tied with a thin rope so that the three pigs could all chew it together, subsequently referred to as “Object.” The Object thus met some of the criteria for an enrichment material, such as chewable and manipulable, but not other criteria like edible and destructible. Human and object familiarization sessions were alternated. Familiarizations started at 28 days of age and ended at 53 days of age. They were divided into two phases for each stimulus: eight sessions in the home pen (from days 29 to 35), and eight sessions in the experimental room (from days 36 to 43 and 49 to 53). In the home pen, each group of three pigs received 10 min sessions twice a day for each stimulus for 4 days. During the same week, all groups were alternately transported to the empty experimental room and remained there for 10 min, once a day for 5 days, to become habituated to the new room. After this habituation, pigs received 10 min sessions of stimulus familiarization in the experimental room, once a day for each stimulus, for 9 days. The same procedure was used for each group of three pen mates, as follows:

- Object: the experimenter came to the gate of the pen holding the object and stood still and quiet for 30 s. Then, she entered the pen to tie the object to the opposite wall with a small rope and went out. From the moment she went out, the object was left for 10 min in the pen. Then the experimenter removed the object.
- Human: the experimenter came to the gate of the pen, holding a 40 cm high stool, and stood still and quiet for 30 s. She then entered to sit on the stool in the pen, close to the opposite. To minimize stress on the first day (day of weaning, day 28) the human engaged in no interaction. From 29 days of age, during each session, she engaged in interactions with each pig [similar to the protocol in (16)]: she held out a hand toward the pig; if the pig did not move away, she tried to touch it; if the pig accepted being touched, she softly stroked it along the body with the palm of her hand; once it accepted being stroked, she scratched it along the body with her fingers. Scratching consisted of rubbing the skin of the pigs with the finger tips and applying more pressure than stroking. In addition, the handler spoke to the pig with a soft voice. The experimenter focused on each pig for 2 min initially and alternately focused her attention during the last 4 min.

The procedure of familiarization was similar in rearing pens and in the experimental room, but the location of the stimulus changed: in rearing pens, the stimulus was attached to the opposite wall from the entrance of the pen. In the experimental room, the stimulus was placed in the center of the room.

### Choice Test

#### Experimental Procedure

**Figure 1:**
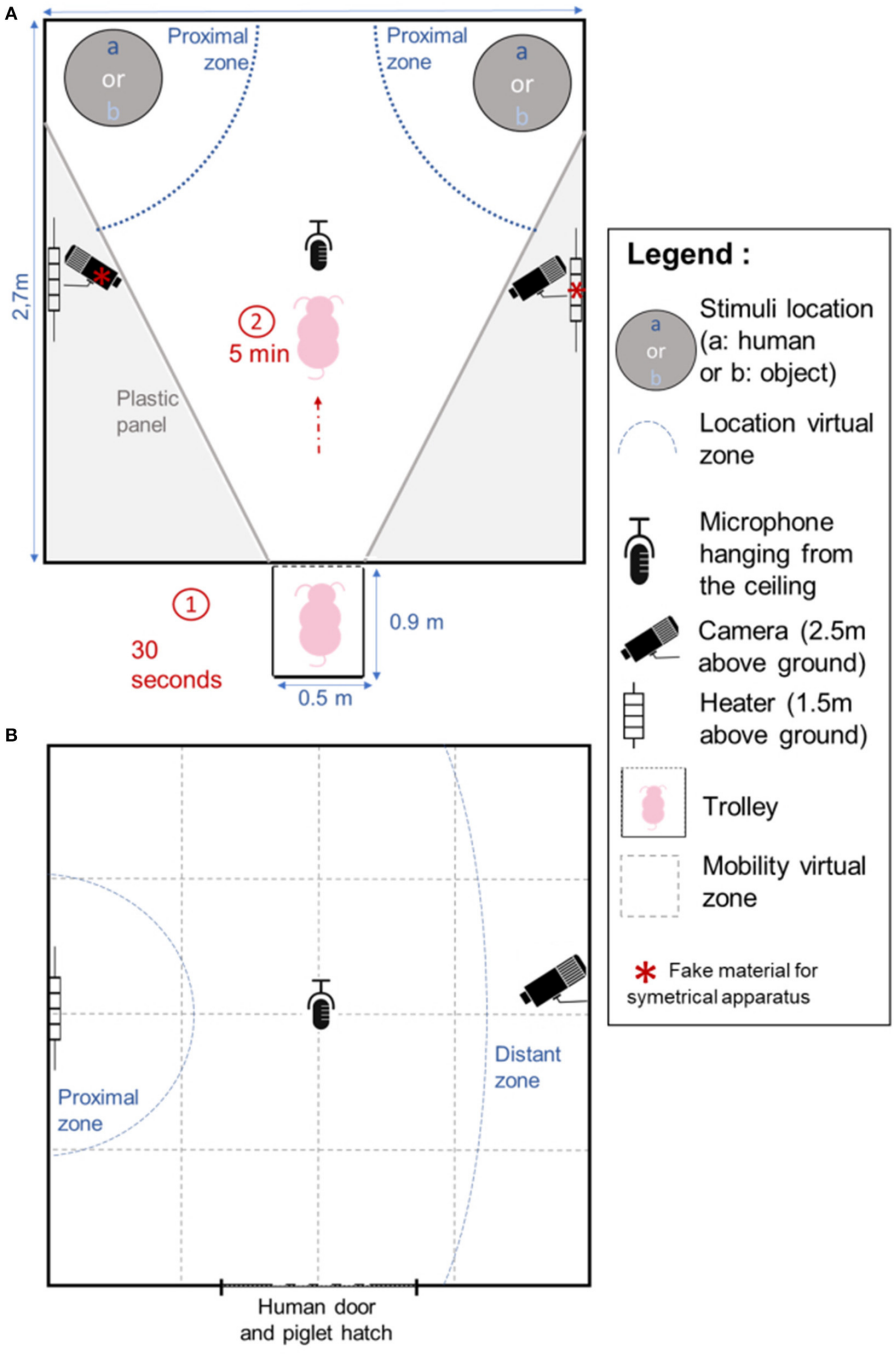
Design of the experimental apparatus for the Choice test **(A)** and the Isolation/Reunion test **(B)**. For the Choice test between Object and Human **(A)**, each pig remained in the trolley for 30 s before entering the room where it was left for 5 min. For the Isolation/Reunion test **(B)**, the pig was left alone for 5 min (Isolation phase), and then remained for 4 min and 30 s (Reunion phase) either alone, with the familiar inanimate Object or with the familiar Human, on different days. Proximal zones: blue solid lines were drawn to identify the zones in which the pig was considered close to the stimulus. The distant zone **(B)** was drawn to identify a zone where the pig was considered distant from the stimulus. Virtual zones were drawn to monitor the location and mobility of the pig in the room (gray dotted lines).

At 47 and 49 days, the pigs were subjected to an individual Choice test between the familiar experimenter and the familiar object, in order to evaluate the potential preference for one of the stimuli. The test took place in the experimental room fitted with a V shaped arena (Figure 1A). The room was, as much as possible, made symmetrical with a false heater and camera, and a homogeneous ground surfacing. On the previous day, pigs were individually left in this room for 5 min in order to become habituated to the room and to being transported alone in a trolley. On the day of the test, the pigs were brought individually to the entrance of the experimental room. Once in front of the experimental room, the hatch to the room and the first hatch of the trolley were opened for 30 s. Since the trolley had another grid hatch, the tested pig could initially see into the experimental room without entering it. The human and the object were already in place at the back of the room (Figure 1A). The caretaker then opened the grid hatch and gently pushed the pig into the room if it had not entered by itself after 2 s. The Choice test lasted 5 min. The experimenter actively called the pig to come to her during the test. If the pig came close, the experimenter petted it, as in the familiarization sessions. This test was done twice, on consecutive days, swapping the sides of the stimuli between days in order to take into account possible bias due to the laterality of the apparatus or the pigs.

#### Behavioral Observations and Analyses

The tests were video recorded by a camera (Bosh, Box 960H-CDD) and a recorder (Noldus Mpeg recorder 2.1., The Netherlands), linked to a LCD monitor (DELL 48 model 1907FPc) which allowed us to visualize the experimental room from an adjacent room. The location of the pigs was monitored directly during the tests and the other behaviors later from videos, both using The Observer XT 14.0 software (Noldus®, The Netherlands). All behaviors recorded are indicated in Table 1 and correspond to the behavior code numbers: 2–7, 11, 14 (restricted to stimulus zone) and 16.

**Table 1:** Ethogram used for the Choice test (1), Isolation/Reunion test (2), and behavioral proximity scores (3).

**Behavior**	**Description**	**Parameter (1: Choice test, 2: Isolation/Reunion test, 3: Behavioral proximity score)**	**References**
**Location of the pig**			
Located in a virtual zone	The pig is considered in a virtual zone when its forelegs and head are in the zone	Number of changes (2)	(1)
Located in stimulus zone	The animal is considered in a zone when its forelegs and head are in the zone	Number of times (1, 3)	(2)
		Mean duration (1, 3)	(3)
		Total time (1, 3)	(4)
		First approach (Human vs. Object)	(5)
		Proportion of time (1)	(6)
		Latency to first entrance (1, 3)	(7)
Located in proximal zone	The pig is considered in the proximal zone when its forelegs and head are in the zone	Total time (2)	(8)
		Latency to first entrance (2)	(9)
Located in distal zone	The pig is considered in the distal zone when its forelegs and head are in the zone	Total time (2)	(10)
**Postures**			
Lying	The pig is lying ventrally or on the flanks	Presence or absence during test	(11)
Standing immobile	The pig is standing still, head up but not oriented toward the entrance door	Total time (3)	(12)
Looking at exit door	The pig is standing still, head turned toward the entrance door	Total time (3)	(13)
Exploring	The pig is sniffing or investigating a part of the environment, wall, or ground, with the snout	Total time (1, 2)	(14)
**Contacts**			
Initiated contacts toward a stimulus	The pig initiates a contact to the stimulus (by head or any body part)	Number of times (3)	(15)
		Total time (1, 3)	(17)
Contact received by the experimenter	The pig is gentled (scratched, stroked) by the experimenter, but did not initiate the contact	Number of times (3)	(18)
		Total time (3)	(19)

Columns describe the name of the behavior, its description, the parameters calculated with it and for which test it was used. A number was assigned to each behavior for reference. The unit for timing is the second, except when it is labeled with a “^*^” for which it is standardized per minute.

### Isolation/Reunion Test

#### Experimental Procedure

At 55, 56, and 57 days of age, pigs were subjected to an Isolation/Reunion test in order to assess their perception of each stimulus and its potential to calm the pigs after a period of stressful isolation (Figure 1B). The test consisted in two phases. The pig was brought individually in a trolley to the experimental room, the hatches were opened and the pig was gently pushed into the room. It was left alone for five min, which defined the “Isolation” phase. Then, one of the stimuli (“Human,” “Object”) or “Nothing” was shown to the pig for 30 s: the door was opened with either: (a) the human standing with the stool, (b) the human standing with the object, or (c) nothing presented. Finally, the second phase named “Reunion” phase occurred and consisted of either (a) presence of the experimenter sitting in the room on a stool and remaining immobile and quiet (“Human” stimulus), (b) presence of the object tied in the room (i.e., “Object” stimulus: the human had to enter the room, install the object, and leave the room), or (c) isolation in the room for 270 s (“No stimulus”). Each pig was thus tested three times, with one test per pig per day. The order of the modalities (reunion with human, object or without stimulus) was randomized between days and between pigs of the same pen.

#### Behavioral Observation and Analyses

The same equipment as for the Choice test was used for the Isolation/Reunion test (see section above), but different behaviors were scored. All behaviors used are indicated in Table 1 and correspond to the behavior code numbers: 1, 8–10, 14.

#### Acoustic Monitoring and Analyses

Vocalizations produced during the Isolation/Reunion test were recorded with a C314 microphone (AKG, Austria) placed in the center of the room at a height of one meter, connected to a MD661MK2 recorder (Marantz, USA). The vocal types were then manually annotated (grunt, squeak, bark, scream, and mixed calls), after visual inspection of spectrograms on Praat® software, by an expert [call types in pigs have been described and grunts are particularly typical low frequency and noisy calls (27, 28)]. Only grunts were subsequently acoustically analyzed as they represented the most frequent call type that constituted a dataset of 5,766 calls. A spectro-temporal analysis was performed with custom-written codes using the Seewave R package (29) implemented in R (30). After a 0.2–8 kHz bandpass filtering (“fir” function), a standardized grunt was detected when the amplitude crossed a 5% amplitude threshold (“timer” function) to measure call duration. After amplitude normalization, the following spectral parameters were calculated [“specprop” function, FFT with Hamming window, window length = 512, overlap = 50%): mean, median, first (Q25) and third (Q75) quartiles, interquartile range (IQR), centroid (all in Hz)]. The grunt dominant frequency (in kHz) was also calculated (“dfreq,” 50% overlapping FFTs, window length = 512), which is the mean over the call duration of the frequencies of the highest level of energy. Parameters measuring noisiness and entropy of the grunt were: Shannon entropy (sh), Spectral Flatness (Wiener entropy, sfm) and Entropy (H) [combining both Shannon entropy and Temporal envelop entropy, length = 512, Hilbert envelop].

### Statistical Analyses

All the statistical analyses were done using R 3.3.3 (30). Synthetic variables were built with Principal Component Analyses (PCA) and models were constructed to test the effect of the factors of interest. Linear or generalized mixed effect models (“lmer” or “glmer” function, “lme4” R package) were used to test two-way interactions between factors and/or continuous covariates; the pig's identity was put as a random factor (repeated measures per pig) in all models, as nesting individuals within pens (nested random effect) did not lead to converging models.

#### Analysis of Choice Test: Spatial Behavior of Pigs

To be able to assess and compare the behaviors during the 5 min of the Choice test and reduce the number of tested variables, a Principal Component Analysis (PCA) was done considering all behaviors directed toward each stimulus [parameters: 2–7, 11, 14 (restricted to stimulus zone) and 16, Table 1] (31). All PCs having an Eigen value above one were kept and constituted three behavioral response scores, which cumulatively explained 81.3 % of the variability (choicePC1−46%, choicePC2−20%, choicePC3−16%, variable loadings, Table 2). The absolute values of each parameter, in several relevant conditions of the study are available in Supplementary Table 1. The three behavioral response scores were used as response variables in linear models testing the interacting effect of the day of the test (two levels: first or second) and the stimulus (two levels Human vs. Object); the position of the human (left or right) was added as a control for choices linked to laterality (model 1). Two additional behaviors were tested as binary variables: the first approach (Human or Object, parameter code 5, Table 1) and whether the pig laid down near one stimulus (presence or absence, parameter code 11, Table 1). To test whether the first approach depended on a stimulus, it was tested in a binomial model (Human or Object) and the effect of the day and the position of the human were put in an additive model (model 2). The number of times a pig laid down in the proximal zone close to a stimulus was tested as a binomial variable (presence vs. absence) and a χ^2^ test was used.

**Table 2:** Variable loadings of the behavioral parameters used in the Principal Component Analysis in the Choice test.

	**Percentage per PC**			**Relative cumulative values**		
	**choicePC1**	**choicePC2**	**choicePC3**	**choicePC1**	**choicePC2**	**choicePC3**
Cumulative inertia	45.8	65.3	81.3	–	–	–
Number of visits in stimulus zone	0.63	60.90	1.84	−2.03	82.86	2.07
Mean duration in stimulus zone	24.19	11.56	1.49	−77.59	−15.73	−1.68
Proportion of time in stimulus zone	23.76	4.38	0.15	−76.21	5.95	0.17
Time spent in contact with the stimulus	22.06	3.22	0.01	−70.77	−4.38	−0.01
Time spent exploring when in stimulus zone	0.22	17.21	37.35	−0.70	23.41	−41.97
Latency to approach zone	0.90	1.62	59.12	2.89	−2.21	−66.44
Total time in zone	28.25	1.11	0.03	−90.64	1.51	−0.04

All Principal components (PCs) having an Eigen value above one were kept to build behavioral response scores. The first line of the table indicates the cumulative inertia explained by the PCs. For each PC, the percentage of (left side) as well as the relative cumulative value (right side) of a given parameter is indicated. Parameters having a percentage above the uniform distribution can be considered as explanatory parameters for a given PC.

#### Analysis of Isolation/Reunion Tests: Spatial and Vocal Behavior of Pigs

##### Behavioral response scores

To be able to have comparable behaviors, between phases and stimuli, and to reduce the number of variables, behaviors were gathered and a PCA was computed (parameter codes: 1, 8–10, 14, Table 1) (31). Only parameters measurable in any condition (phase of the test and type of reunion) were kept and the percentage of explained variance maximized. All PCs having an Eigen value above one were kept and constituted three behavioral response scores, which cumulatively explained 82% of the variability (IsoReuPC1−32%, IsoReuPC2−39%, IsoReuPC3−11%, variable loadings, Table 3). The absolute values of each parameter, in relevant groups of the study are available in Supplementary Table 2.

**Table 3:** Variable loadings of the behavioral parameters used in the Principal Component Analysis in the Isolation/Reunion test.

	**Percentage per axis**			**Relative cumulative values**		
	**IsoReuPC1**	**IsoReuPC2**	**IsoReuPC3**	**IsoReuPC1**	**IsoReuPC2**	**IsoReuPC3**
Cumulative inertia	31.5	70.5	82	–	–	–
Time spent standing immobile	22.89	3.49	6.33	−50.52	−5.20	−7.82
Time spent looking at exit door	2.98	37.10	13.93	−6.57	−55.32	−17.21
Time spent in proximal zone	6.69	5.34	47.46	14.77	7.96	−58.61
Time spent in distal zone	27.34	2.16	3.26	−60.33	3.22	4.03
Number of virtual zone changes	21.90	5.86	19.76	−48.32	8.74	−24.39
Time spent exploring the room	12.00	25.83	2.60	−26.47	38.52	3.21
Latency to enter proximal zone	6.21	20.22	6.66	−13.69	−30.15	8.22

*All Principal components (PCs) having an Eigen value above one were kept to build behavioral response scores. The first line of the table indicates the cumulative inertia explained by the PCs. For each PC, the percentage of (left side) as well as the relative cumulative value (right side) of a given parameter is indicated. Parameters having a percentage above the uniform distribution can be considered as explanatory parameters for a given PC. Parameters quantifying total duration or numbers were standardized per minute*.

##### Acoustic scores

To be able to compare the spectro-temporal structure of grunts, two scores were built. The duration of grunts was log transformed and used as a temporal score (linear distribution). For spectral analysis, parameters previously extracted were gathered in a PCA to be able to monitor which parameters load the same way and build an acoustic score. Only one PC had an Eigen value above one, explained 83% of the variability and was named “Acoustic spectral score (PCac.).” The absolute values of each parameter, in relevant groups of the study, are available in Supplementary Table 3.

##### Statistical models

The three behavioral response scores (IsoReuPC1, IsoReuPC2, IsoReuPC3) and the two acoustic scores [PCac. and log(duration)] were used as response variables in a linear model testing (i) the two-way interaction between the type of reunion (Human/Object/No stimulus) and the phase of the test (Isolation/Reunion), (ii) the two-way interaction between the day of the test (1/2/3) and the phase, (iii) the day of the test and the type of reunion (model 3).

#### Analyses of Predictors for Vocal Expression During the Reunion With the Stimulus

The aim of this analysis was to search for the best predictors of vocal dynamic and grunt acoustic features, in the presence of the human or the object. For this analysis, only the dataset containing the reunion phases with the Human or the Object were used, extracted from the Isolation/Reunion test.

##### Monitoring spatial proximity toward the stimulus and time during the test

The location of the pig in the room was divided into two categories: when the pig was located in the proximal zone (“Close”) and when the pig was located elsewhere (“Away”) to build a two level factor named “Location.” This parameter allowed us to track for *spatial proximity* toward the stimulus. Each period of time that the pig was Close or Away was considered as a time interval. Each time interval was numbered to track the rank of the interval during the test and the “Interval index” variable was created (e.g., Close1, Away2, Close3…).

##### Building behavioral proximity scores toward the stimulus

Using the behavioral observations during the Choice test, *behavioral proximity scores* reflecting the closeness and exploration toward each stimulus (parameters: 2–4, 7, 12–13, 15–18, Table 1) were built using two PCAs (one per type of stimulus). Only the first principal component was kept in each PCA (HproxPC1 and OproxPC1) to be used as behavioral proximity score for a specific stimulus (variable loadings Table 6). Only scores from day 1 were used, to minimize habituation effects that could occur on day two. For the human, HproxPC1 explained 63% of data variability and, for the object, OproxPC1 explained 47%. For further analyses, the score toward each stimulus was matched accordingly to the type of reunion the pig was experiencing (Human vs. Object): when reunited with the human, the behavioral proximity score toward the human (HproxPC1) was used, whereas when reunited with the object, the behavioral proximity scores toward the object (OproxPC1) was used.

##### Model selection: searching for the best predictors of vocal expression

During the reunion with a stimulus (Human or Object), several variables could explain the vocal expression of pigs: the day of the test (3 levels), the time during the test (index of the time interval in a zone as a continuous variable), the spatial proximity of the pig toward the stimulus (two levels: close to or away from the stimulus), the behavioral proximity of the pig toward the stimulus (continuous behavioral proximity score) or interactions between the type of stimulus and the location, between the type of stimulus and the behavioral proximity toward the stimulus or between the type of stimulus and the time during the test. To search for the best predictors of vocal expression, five acoustic variables were used as response variables. Three variables were linear: the acoustic spectral score PCac., the duration of grunt [log(grunt duration)] and the grunt rate (number of grunts per second, calculated when the number of grunts per interval was above three (186 intervals out of 286 intervals). Two non-linear variables were used: the total number of grunts (Poisson distribution), the number of times grunts were produced in series (Binomial distribution), i.e., when more than one grunt was produced in a given interval. Indeed, since we used only intervals containing at least three grunts to calculate the grunt rate, we needed to ensure we were not missing information on intervals containing fewer grunts, so we used the occurrence of one-grunt intervals to counteract the effect of interval selection.

A full model, describing the experimental design, was built as follows (“lmer” or “glmer” function of “lme4” R package): *Model 4* = *Response variable* ~*day* + *stimulus* + *location* + *Z interval index* + *Z behavioral proximity score* + stimulus^*^location + stimulus^*^Z behavioral proximity score + stimulus^*^Z interval index + location^*^Z behavioral proximity score +*(1|individual)*. To increase interpretability, all continuous variables (interval index and behavioral proximity scores) were scaled, so the Z score is presented every time (32), the individual was put as a random factor to take into account multiple tests on the same pig. On this full model, a model selection was performed with the “dredge” function of the “MuMIn” R package (33), which compares all possible models built using subsets of the initial explanatory variables of the full model, including the null model. Models were compared using Akaike Information Criteria corrected for small sample size (AICc). Significant models were selected when delta AICc was below two (34), the weight of remaining explanatory variables was evaluated by calculating the presence or absence of the term in the remaining models (“importance” function). It should be noted that for the occurrence of one grunt intervals (Binomial model), no significant models were selected since the null model was contained in the best selected models (AICc <2). Although not mentioned in the results section, the model selection table is available (Supplementary Table 7).

#### Tests and Validation of All Models and Model Selection

All linear models were validated by visual inspection of the symmetrical and normal distribution of the residuals [“plotresid” in “RVAideMemoire” R package (35)]. For generalized models, overdispersion was tested using the “overdisp.glmer” function (“RVAideMemoire” R package); when overdispersed, a correction with the line number as random factor was used.

Anovas were computed on models to test for significant effects of explanatory variables (“car” R package), effects were considered significant when the *p*-value was below 0.05. Model estimates and pairwise *post hoc* tests were computed using Tukey correction for multiple testing [“lsmeans” R package (36) (models 1–3)]. A complete report of statistics is available as Supplementary Tables 4–6.

For the model selection (model 4), the analysis does not give *p*-values but rather a subset of significant models and weight of predictors. A model averaging step (“model.avg” function) gives the estimates of each of the predictors. The best predictors were the ones with a weight of one, meaning they were consistently present in all selected models. A complete report of all best equivalent models is available in Supplementary Table 7.

## Results

### Choice Test Between Human and Object

The PCA allowed us to extract three behavioral response scores, respectively, choicePC1, choicePC2, and choicePC3 that explained cumulatively 81% of data variability (Table 2). Only the statistical analyses on PCs are presented in the result section, but Supplementary Table 2 describes each behavioral parameter depending on the experimental conditions.

**Figure 2:**
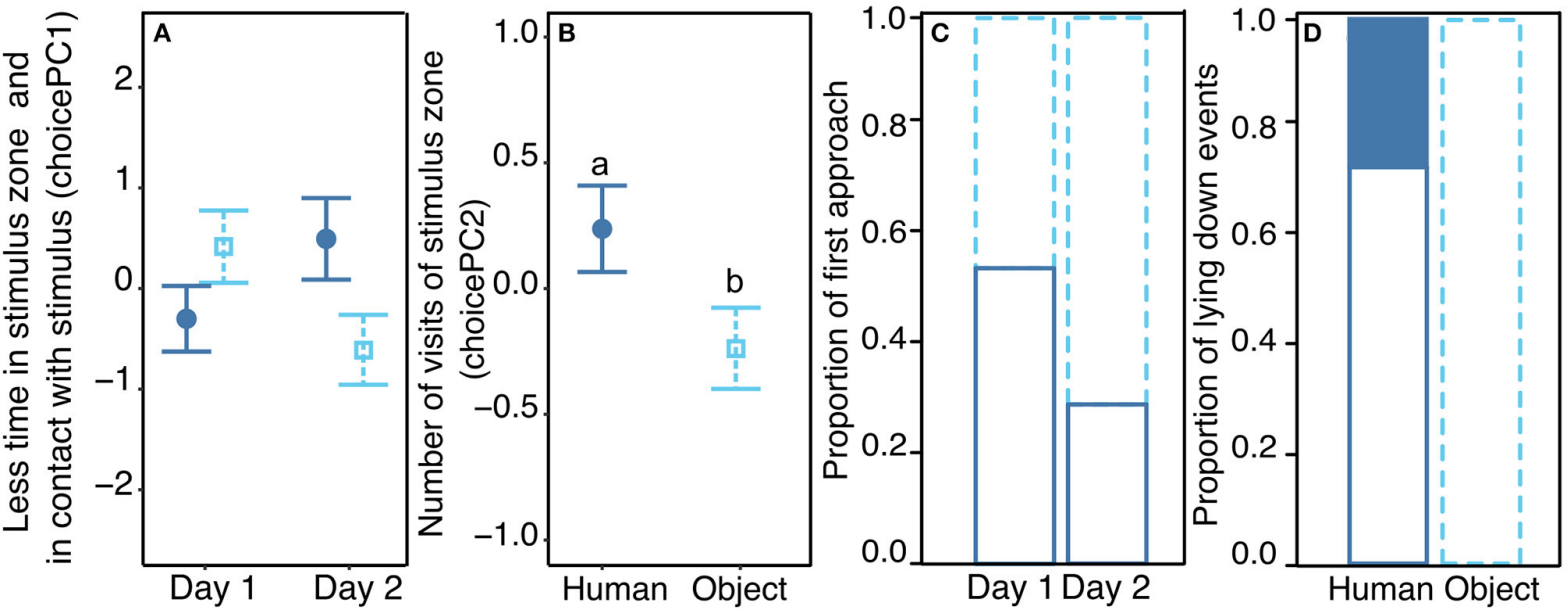
Behavioral response in the Choice test. Mean (±se) of the two behavioral response scores from the PCA analysis, choicePC1 **(A)** and choicePC2 **(B)**, toward the two possible stimuli: either Human (filled dark blue circles) or Object (empty light blue squares). **(A)** Significant interaction between the stimulus and the day of the test but no differences revealed between groups in *post hoc* tests. **(B)** Significant effect of the stimulus on choicePC2. **(C)** first approach of the pig toward one of the stimuli either human (solid dark blue bars) or object (dotted light blue bars), indicated as proportion of the 24 pigs tested twice (day 1 and day 2). **(D)** Proportion of times that pigs laid down during the test either in the human zone or in the object zone. Different letters indicate significantly different groups (*p* < 0.05). All model estimates, anova tables, and results of *post ho*c tests are available in Supplementary Tables 4–6. Description of each behavioral parameter depending on experimental conditions is available in Supplementary Table 1.

On the first behavioral response score (choicePC1, 45.8%), the mean duration in the stimulus zone, the proportion of time spent in the stimulus zone, the time spent in contact with the stimulus and the total time spent in the zone loaded negatively. Statistics revealed a significant effect of the interaction between the type of stimulus and the day of the test ($\chi_{1}^{2}$ = 6.3, *p* = 0.012), but *post hoc* tests did not show any difference between groups (pairwise tests with Tukey correction, |t.ratio| <2.2, *p* > 0.15, Figure 2A). On the second behavioral response score (choicePC2, 19.5%), the number of visits in the stimulus zone loaded positively. Statistics showed no interaction between the type of stimulus and the day of the test ($\chi_{1}^{2}$ = 0.7, *p* = 0.4), a trend for an effect of the day ($\chi_{1}^{2}$ = 3.3, *p* = 0.07) and a main effect of the type of stimulus: PC2 was higher when considering the human zone compared to the object zone ($\chi_{1}^{2}$ = 7.3, *p* = 0.007, Figure 2B). On the third behavioral response score (choicePC3, 16%), the time the pig spent exploring the stimulus zone and the latency to approach the stimulus zone loaded negatively. Statistics showed no effect of explanatory variables on choicePC3 (Stimulus: $\chi_{1}^{2}$ = 1.5, *p* = 0.2, Day: $\chi_{1}^{2}$ = 0.6, *p* = 0.5). We examined the number of times the human zone or the object zone was first approached by the pig during the test. Statistics on this binary variable showed a trend for an effect of the day of the test: pigs tended to first approach the object zone more often on the second day than on the first day of the test ($\chi_{1}^{2}$ = 3.4, *p* = 0.06, Figure 2C). Finally, we counted the number of times pigs laid down near a stimulus zone; a χ^2^ test showed a significantly different distribution of occurrences of this behavior, which only occurred in the human zone (by nine individuals out of 24) and never in the object zone ($\chi_{1}^{2}=$ 12.8, *p* < 0.001, Figure 2D). The position of the human in the room (left or right side) was included in all models and never showed a significant effect (see Supplementary Table 4 for full report).

### Isolation/Reunion Test

#### Variation in Pigs' Behavior When They Are Reunited With a Human or An Object

For the Isolation/Reunion test, a PCA allowed us to extract three behavioral response scores, respectively IsoReuPC1, IsoReuPC2 and IsoReuPC3 that explained cumulatively 82% of data variability (Table 3). Only the statistical analyses on PCs are presented in the results section, but Supplementary Table 2 describes each behavioral parameter depending on the experimental conditions.

**Figure 3:**
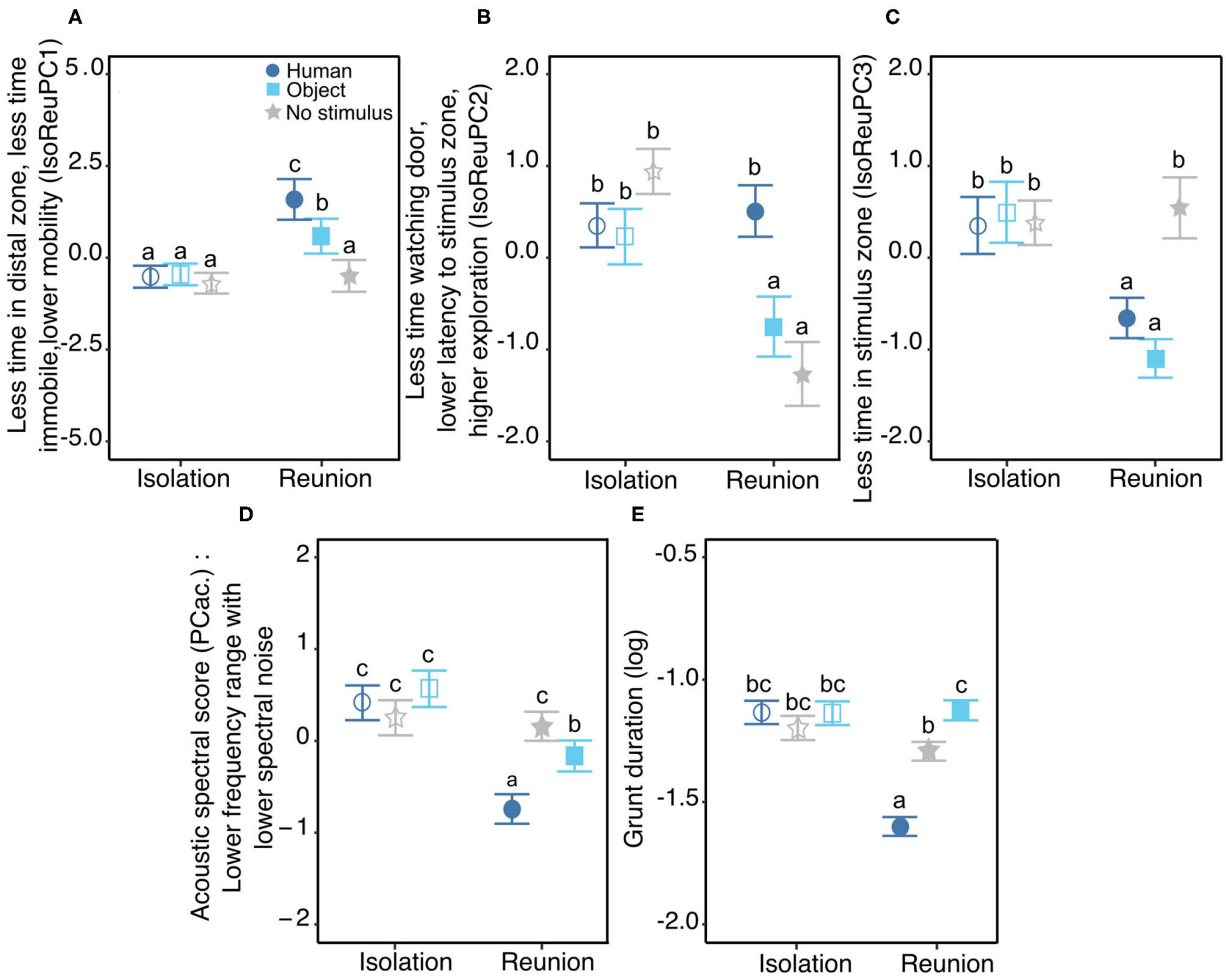
Behavioral **(A–C)** and vocal **(D,E)** responses to the Isolation/Reunion test. Mean (±se) of behavioral and acoustic scores, according to the stimulus (Human = dark blue circles, Object = light blue squares, No stimulus = gray stars), the phase of the test (Isolation = empty symbols or Reunion = filled symbols) and/or the day of the test (day 1, 2, or 3). **(A–C)** significant interaction between the type of reunion and the phase of the test for the three behavioral response scores IsoReuPC1, IsoReuPC2, IsoReuPC3, respectively. **(D,E)** significant interaction between the type of reunion and the phase of the test for the two acoustic scores: the acoustic spectral score **(D)** and the logarithm of grunt duration **(E)**. Different letters show significantly different groups (*p* < 0.05). All model estimates, anova tables, and results of *post hoc* tests are available in Supplementary Tables 4–6. Description of each behavioral and each acoustic parameter depending on experimental conditions is available in Supplementary Tables 2, 3, respectively.

On the first behavioral response score (IsoReuPC1, 31.5%), the time spent immobile, the time spent in the distal zone and the number of changes of virtual zone negatively loaded. Statistics revealed a significant interaction between the type of reunion and the phase of the test ($\chi_{2}^{2}$ = 16.6, *p* < 0.001, Figure 3A). During the isolation phase, no significant difference was found between groups (pairwise comparisons human/object/no stimulus, |t.ratio| <0.7, *p* > 0.9), whereas during the reunion phase the three type of reunion differed significantly in PC1 values (human vs. object: t.ratio = 3.1, *p* = 0.03, human vs. no stimulus: t.ratio = 6.3, *p* < 0.001, object vs. no stimulus: t.ratio = 3.3, *p* = 0.02). Furthermore, the reaction to each reunion type did not have the same magnitude. When pigs were not reunited with a stimulus, statistics did not show differences between the isolation and the reunion phases (isolation vs. reunion without stimulus: t.ratio = 0.6, *p* > 0.9), whereas when reunited with a stimulus, IsoReuPC1 showed a significant increase that was stronger with the human (isolation vs. reunion, t.ratio = −6.3, *p* < 0.001) than with the object (isolation vs. reunion, t.ratio = −3.2, *p* < 0.03).

On the second behavioral response score (IsoReuPC2, 39%), the time spent exploring the room loaded positively and the time spent looking at the entrance door and the latency to enter the proximal zone of the stimulus loaded negatively. A significant interaction was found between the type of reunion and the phase of the test ($\chi_{2}^{2}$ = 41.5, *p* < 0.0001, Figure 3B). During the isolation phase, no significant difference was found between groups (pairwise comparison human/object/no stimulus, |t.ratio| <2.7, *p* > 0.08), whereas during the reunion phase the two types of stimuli differed significantly (human vs. object: t.ratio = 4.9, *p* < 0.001), as well as the reunion with the human compared to no stimulus (t.ratio = 6.8, *p* < 0.001), but no difference was found when comparing reunions with the object or without stimulus (t.ratio = 2.0, *p* = 0.37). The reaction to the three types of reunions also differed: from isolation to reunion phase, no difference was found in IsoReuPC2 when pigs were reunited with the human (t.ratio = −0.6, *p* = 0.9), whereas PC2 decreased significantly when pigs were reunited with the object or without stimulus (object: t.ratio = 3.8, *p* = 0.003, no stimulus: t.ratio = 8.5, *p* < 0.001).

On the third behavioral response score (IsoReuPC3, 11.5%), the time spent in the stimulus zone loaded negatively. Statistics showed a significant interaction between the type of reunion and the phase of the test on IsoReuPC3 ($\chi_{2}^{2}$ = 36.4, *p* < 0.001, Figure 3C). During the isolation phase, no significant difference was found between groups (pairwise comparison human/object/no stimulus, |t.ratio| <0.7, *p* > 0.9). During the reunion phase, IsoReuPC3 differed significantly for pigs being reunited without stimulus compared to being reunited with a stimulus (human vs. no stimulus: t.ratio = −5.7, *p* < 0.001, object vs. no stimulus: t.ratio = −7.8, *p* < 0.001), but IsoReuPC3 did not differ between the two types of stimuli (human vs. object: t.ratio = 2.1, *p* = 0.3). The reaction to the three types of reunions also differed: from isolation to reunion phase, no difference was found in IsoReuPC3 when pigs were not reunited with a stimulus (reunion with no stimulus: t.ratio = −0.8, *p* = 0.9), whereas IsoReuPC3 decreased significantly when pigs were reunited with the object or with the human (object: t.ratio = 7.6, *p* < 0.001, human: t.ratio = 4.8, *p* < 0.001).

The day of the test did not show any effect on IsoReuPC2 and IsoReuPC3 ($\chi_{2}^{2}$ = 0.9, *p* = 0.6, $\chi_{2}^{2}$ = 0.2, *p* = 0.9, respectively) but was significantly higher for IsoReuPC1 from day 1 to day 3 ($\chi_{2}^{2}$ = 10.1, *p* = 0.007, Supplementary Figure **1A**). *Post hoc* testing showed the differences in IsoReuPC1 were progressive over days (pairwise comparison, day 1 vs. day2: t.ratio = −2.4, *p* = 0.05, day 1 vs. day 3: t.ratio = −3.0, *p* = 0.009, day 2 vs. day3: t.ratio = −0.6, *p* = 0.79).

#### Pigs' Grunt Acoustic Features When They Are Reunited With a Human, An Object Or Without Stimulus'

All 5,766 grunts produced during the test were analyzed using two acoustic scores: the logarithm of grunt duration and a spectral score. This spectral score is the first principal component of a PCA containing frequency and noise parameters of the calls (acoustic spectral score PCac., variable loading Table 4): the greater the score, the lower the frequency and the lower the spectral noise in the grunt. Only the statistical analyses on scores (temporal and spectral) are presented in the results section, but Table 5 and Supplementary Table 3 describe each acoustic parameter depending on the experimental conditions.

**Table 4:** Variable loadings of the parameters used in the Principal Component Analysis to build a spectral acoustic score.

	**Acoustic spectral score (PCac.)**	
	**Percentage on axis**	**Relative cumulative values**
Cumulative inertia	83.496	–
Mean	14.820	−98.992
Centroid	14.820	−98.992
Inter Quartile Range	13.624	−91.003
Spectral Flatness (sfm)	14.492	−96.802
Shannon index (sh)	14.398	−96.172
Entropy	13.797	−92.159
Mean Dominant frequency	1.193	−7.967
Spectral Standard Deviation (sd)	12.858	−85.885

*All Principal components (PCs) having an Eigen value above one were kept to build an acoustic spectral response score. The first line of the table indicates the cumulative inertia explained by the PCs. Only one PC was kept. The table indicates the percentage of (left side) as well as the relative cumulative value (right side) of a given parameter. Parameters having a percentage above the uniform distribution can be considered as explanatory parameters for a given PC*.

**Table 5:** Raw values for acoustic parameters used in acoustic scores for significant interaction groups (stimulus and phase of test interaction).

			**Mean (Hz)**		**Centroid (Hz)**		**Mean Dominant Frequency (KHz)**	
**Stimulus**	**Phase of Test**	**N**	**Mean**	**sd**	**Mean**	**sd**	**Mean**	**sd**
Human	Isolation	673	975.6	240.9	975.6	240.9	0.295	0.026
Human	Reunion	1,302	1135.0	240.9	1135.0	357.4	0.307	0.065
No stimulus	Isolation	775	1002.8	240.9	1002.8	280.9	0.295	0.037
No stimulus	Reunion	1,286	1018.4	240.9	1018.4	307.5	0.301	0.039
Object	Isolation	755	973.9	240.9	973.9	294.1	0.293	0.040
Object	Reunion	975	1042.7	240.9	1042.7	292.9	0.299	0.040
			**Inter Quartile Range (Hz)**		**spectrum standard deviation**		**Call duration (s)**	
Human	Isolation	673	636.4	504.0	1473.8	254.9	0.379	0.222
Human	Reunion	1302	941.1	768.4	1603.7	296.6	0.251	0.185
No stimulus	Isolation	775	719.7	617.1	1490.4	274.3	0.362	0.219
No stimulus	Reunion	1286	749.9	675.9	1493.4	281.7	0.333	0.207
Object	Isolation	755	650.3	623.3	1461.8	274.9	0.385	0.241
Object	Reunion	975	776.6	633.9	1524.8	266.5	0.385	0.222
			**Shannon entropy**		**Spectral Flatness**		**Entropy**	
Human	Isolation	673	0.651	0.067	0.270	0.087	0.501	0.049
Human	Reunion	1,302	0.686	0.079	0.319	0.114	0.517	0.058
No stimulus	Isolation	775	0.655	0.072	0.277	0.098	0.504	0.053
No stimulus	Reunion	1,286	0.660	0.077	0.280	0.104	0.506	0.058
Object	Isolation	755	0.647	0.074	0.267	0.100	0.498	0.054
Object	Reunion	975	0.669	0.074	0.291	0.098	0.516	0.054

*The number of vocalizations per group and the mean and standard deviation (sd) are indicated. A more complete table is available in the Supplementary Table 3. No statistics were run on these parameters (see Methods section)*.

Concerning the spectral acoustic score (PCac.), a significant interaction was found between the type of reunion and the phase of the test ($\chi_{1}^{2}$ = 45.1, *p* < 0.001, Figure 3D). During the isolation phase, no difference was found between groups (pairwise comparison during isolation, human/object/no stimulus: |t.ratio| <1.9, *p* > 0.4), whereas during the reunion phase significant differences were found between groups (pairwise comparisons during reunion, human vs. object: t.ratio = −4.9, *p* < 0.001, human vs. no stimulus: t.ratio = −9.2, *p* < 0.001, no stimulus vs. object: t.ratio = 3.7, *p* = 0.003). Furthermore, the reaction to each of the reunion types did not have the same magnitude of change. When pigs were subjected to another isolation, statistics did not show differences between the isolation and the reunion phase (t.ratio = 0.03, *p* = 1), whereas when reunited with a stimulus PCac. showed a significant decrease that was stronger with the human (t.ratio = 9.3, *p* < 0.001) than with the object (t.ratio = 5.3, *p* < 0.001). Statistics also showed a significant interaction between the type of reunion and the day of the test ($\chi_{1}^{2}$ = 26.8, *p* < 0.001) but *post hoc* tests revealed no significant pairwise comparisons (|t.ratio| <1.6, *p* > 0.8, see Supplementary Tables 4–6 and Supplementary Figure 1B).

Grunt duration showed a significant interaction between the type of reunion and the phase of the test ($\chi_{2}^{2}$ = 210.1, *p* < 0.001, Figure 3E). During the isolation phase, no difference was found between groups (pairwise comparison during isolation, human/object/no stimulus: |t.ratio| <2.6, *p* > 0.09), whereas during the reunion phase significant differences were found between groups (pairwise comparisons during reunion, human vs. object: t.ratio = −19.5, *p* < 0.001, human vs. no stimulus: t.ratio = −16.7, *p* < 0.001, no stimulus vs. object: t.ratio = −3.9, *p* = 0.003). The reaction to each of the reunion types also differed. When pigs were subjected to another isolation or reunited with the object, statistics did not show differences between the isolation and the reunion phase (pairwise comparisons isolation vs. reunion object/ no stimulus: |t.ratio| <0.6, *p* > 0.6), whereas when reunited with the human, grunt duration decreased significantly (pairwise comparison isolation vs. reunion, human: t.ratio = 9.3, *p* < 0.001). Finally, statistics also revealed a significant main effect of the day of the test ($\chi_{2}^{2}$ = 20.0, *p* < 0.001): grunt duration decreased as the day of the test increased, especially between the first 2 days (pairwise comparisons, day 1 vs. day 2: t.ratio = 3.9, *p* < 0.001, day 1 vs. day 3: t.ratio = 2.6, *p* = 0.03, day 2 vs. day 3: t.ratio = −1.2, *p* = 0.4, Supplementary Figure 1C).

### Effect of Proximity to Stimulus on Vocal Expression

The following four acoustic variables: total number of grunts, grunt rate, duration of grunts [log(grunt duration)] and spectral acoustic score (PCac.) may be predicted by the context (the type of stimulus), the spatial proximity to the stimulus (location in the room), variables independent from the stimuli (day, time during the test, described by the interval index) or the experience pigs previously had with the stimuli. To quantify the experience pigs had with each stimulus (closeness and exploration), behavioral proximity scores resulting two from principal component analyses were built (Table 6) and one was selected per type of reunion: “behavioral proximity score” corresponded to the opposite sign of HproxPC1/OproxPC1 (respectively, for reunion with the human or the object) and was positively correlated with the time spent in contact with and near the stimulus. After model comparison and selection of the best equivalent models, the weight of predictors as well as the estimates of the averaged resulting model were calculated (Tables 7, 8, respectively, full selected models in Supplementary Table 7).

**Table 6:** Variable loading of PCA describing pig-stimulus behavioral proximity.

	**Human proximity score (HproxPC1)**	**Object proximity score (OproxPC1)**
	**Percentage on axis**	
Cumulative inertia (%)	63.075	46.988
Latency to approach stimulus zone	1.304	0.031
Number of times in stimulus zone	1.859	0.204
Mean duration in stimulus zone	13.311	26.097
Total time in stimulus zone	18.363	31.575
Total time all contacts (human)	16.822	–
Total number of all contacts (human)	16.191	–
Total time of initiated contacts toward stimulus	15.834	30.142
Total number of initiated contacts toward stimulus	16.318	11.951
	**Relative cumulative values**	
Latency to approach stimulus zone	6.578	0.087
Number of times in stimulus zone	−9.378	−0.575
Mean duration in stimulus zone	−67.165	−73.574
Total time in stimulus zone	−92.657	−89.020
Total time all contacts	−84.882	–
Total number of all contacts	−81.699	–
Total time of initiated contacts toward stimulus	−79.899	−84.978
Total number of initiated contacts toward stimulus	−82.342	−33.693

*Only the first Principal component was kept to create a score to be used as an explanatory continuous variable. The first line of the table indicates the cumulative inertia explained by the selected PC, the percentage of (above) as well as the relative cumulative value (below) of a given parameter is indicated. Parameters having a percentage above the uniform distribution can be considered as explanatory parameters for the PC. Behavioral parameters used to build these scores were extracted from the first Choice test. For statistics, the behavioral proximity score toward each stimulus was matched according to the type of reunion the pig was experiencing (Human vs. Object): when reunited with the human, the behavioral proximity score toward the human (HproxPC1) was used, whereas when reunited with the object, the behavioral proximity score toward the object (OproxPC1) was used*.

**Table 7:** Weight of predictors for each response variable.

	***N***	**Stim**	**Day**	**Loc**	**-Behav Prox**	Stim *Day	Stim *Loc	Stim *-Behav Prox	Loc *-Behav Prox	**Int Index**	Stim *Int index
Total Number of grunt-*Poisson*	2	1.00	1.00	1.00	1.00	–	1.00	1.00	0.47	NA	NA
Grunt rate (log)	2	1.00	–	–	0.30	–	–		–	–	–
Grunt duration (log)	6	1.00	1.00	1.00	1.00	–	1.00	0.24	0.25	1.00	0.51
Acoustic spectral score (PC1ac.)	9	1.00	1.00	1.00	1.00	0.36	1.00	0.44	0.13	1.00	0.24

The number of equivalent best models (N), and each term of the full initial model are indicated in columns: “Stim” for stimulus type, Day, “Loc” for location in the room (Close to or Away from the stimulus), “Int. Index” for interval index, “Behav Prox” for behavioral proximity score, as well as relevant interactions between explanatory variables. Only weights different from zero are indicated. For the total number of grunts, since the variable is a sum of all intervals per day, location, individuals and type of stimulus, the interval index was not included in the full model and referred as “NA.” The best predictors are the one consistently appearing in all equivalent selected models so the ones having a weight of “1”.

**Table 8:** Estimates (standard error) of terms contained in the equivalent best selected models.

	**(Intercept)**	**Stim. (Object)**	**Day (2)**	**Day (3)**	**Loc. (Close)**
Total Number of grunt *(Poisson)*	2.770 (0.166)	0.048 (0.074)	0.202 (0.068)	0.010 (0.072)	0.064 (0.060)
Grunt rate (log)	−1.563 (0.133)	−0.428 (0.092)	–	–	–
Grunt duration (log)	−1.496 (0.075)	0.390 (0.042)	−0.125 (0.041)	−0.035 (0.044)	−0.272 (0.039)
Acoustic spectral score (PC1)	−0.222 (0.262)	0.503 (0.276)	−0.660 (0.261)	−0.506 (0.274)	0.191 (0.165)
	**Int. Index**	**-Behav Prox**	Stim. (object) * Loc. (Close)	Day (2) * Stim. (Object)	Day (3) * Stim. (Object)
Total Number of grunt *(Poisson)*	NA	0.053 (0.055)	−0.501 (0.098)	–	–
Grunt rate (log)	–	0.021 (0.031)	–	–	–
Grunt duration (log)	0.108 (0.018)	−0.056 (0.026)	0.223 (0.060)	–	–
Acoustic spectral score (PC1)	0.285 (0.074)	−0.225 (0.109)	−0.644 (0.255)		
	Stim. (object) * Int. Index	Stim. (object) * –Behav Prox	Loc. (Close) * –Behav Prox		
Total Number of grunt *(Poisson)*	NA	0.466 (0.101)	−0.038 (0.025)		
Grunt rate (log)	–	–	–		
Grunt duration (log)	−0.027 (0.019)	0.014 (0.020)	0.007 (0.009)		
Acoustic spectral score (PC1)	−0.041 (0.053)	−0.143 (0.106)	−0.009 (0.027)		

*When the term is a factor, the estimate is indicated for one level and the absolute value has to be calculated using the estimate of the intercept, the level compared is indicated. When the term is a continuous covariate, the estimate of the slope is indicated, notice that to increase interpretability, all continuous variables were scaled so the value is for the Z score. “–”the term was not selected in the best equivalent model. “NA” the term was not in the full model prior to the model selection*.

**Figure 4:**
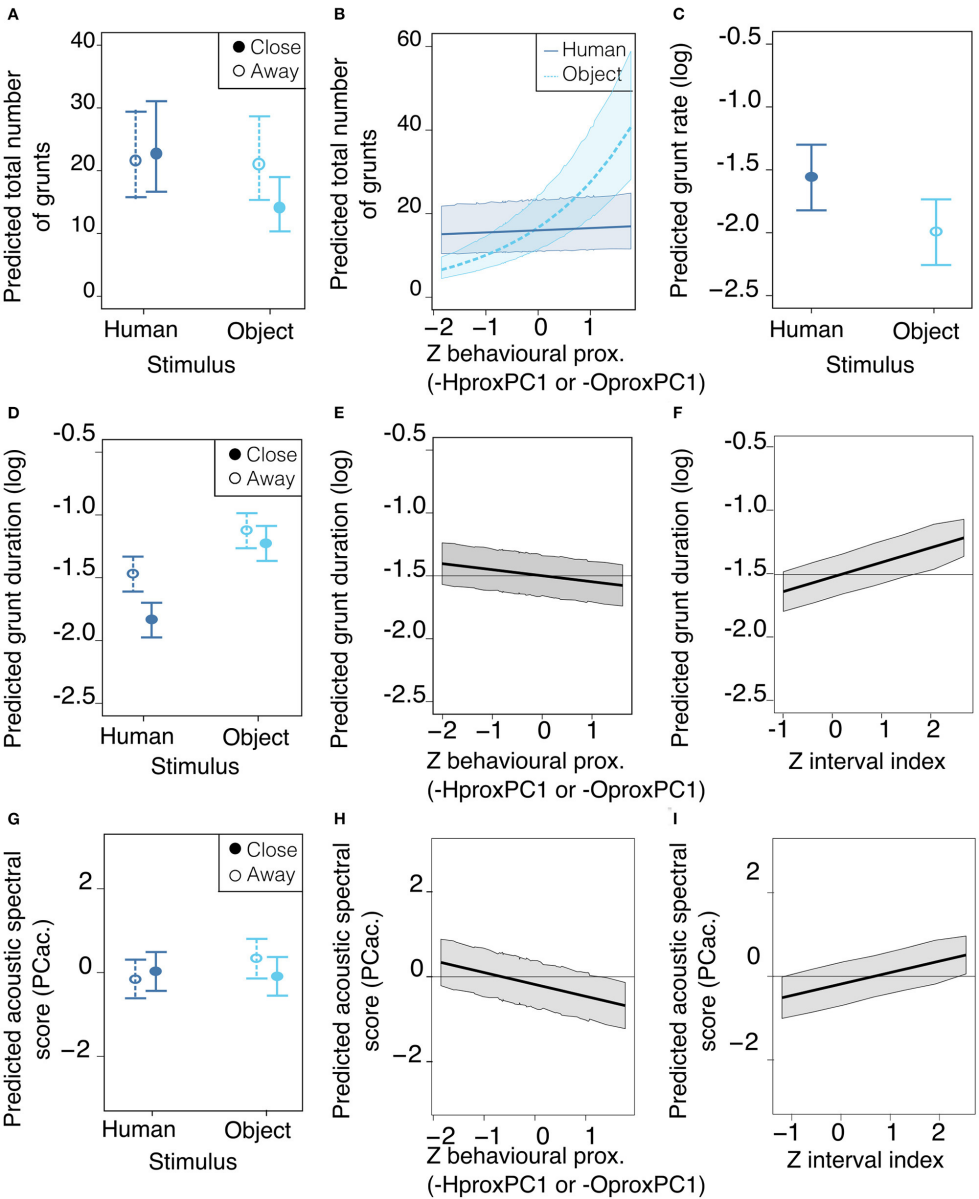
Mean estimates and 95% confidence interval of best predictors of vocal expression: number of grunts **(A,B)**, grunt rate **(C)**, grunt duration **(D–F)** and acoustic spectral score PCac. **(G–I)**, depending on stimulus cyan for object, dark blue for human, location in the room, behavioral proximity and time during the test. Best predictors are represented for illustrating the range and directions of effects. Location **(A,D,G)**: whether the pig was located close to the stimulus (solid lines) or away from it (dotted lines). Behavioral proximity score **(B,E,H)** was scaled for the statistical analysis so the Z score is represented (see composition of scores in Table 6). **(A–D,G)** Type of stimulus: whether the reunion was with the human (dark blue solid circles and lines) or the object light blue empty circles and dotted lines. Time during the test **(F,I)** is quantified by the interval index during the test and is scaled, so the Z score is represented. Plots were generated using the averaged best model resulting from the model selection (models having delta AICc below 2 and predictors having a weight of 1), for which the estimates (se) are in Table 8, the full selection model table is available in Supplementary Table 7.

The model selection showed the total number of grunts was predicted by the interactions between the type of stimulus and the location of the pig in the room, as well as the interaction between the type of stimulus and the behavioral proximity score (Table 6). Thus, a lower number of grunts was likely to occur when the pig was reunited with the object, and spatially close to it (Figure 4B). In addition, when reunited with the object, the higher the behavioral proximity score (-OproxPC1), the higher the probability of producing more grunts (Figure 4A), but this was not the case with the human. Concerning grunt rate, the type of stimulus was the only consistent predictor (Table 7): the rate of grunt was higher when pigs were reunited with the human, than with the object (Figure 4C).

Considering the acoustic structure of grunts (duration and spectral acoustic score PCac.), both descriptors were best predicted by the interaction between the location in the room and the type of stimulus, the behavioral proximity score, the interval index and the day (Table 7). The probability of having shorter grunts was higher when reunited with the human and close to her (Figure 4D). In addition, the higher the behavioral proximity score, the higher the probability of having shorter grunts (Figure 4E). The probability of having longer grunts increased as the time of the test increased (interval index, Figure 4F) with no interaction with the type of stimulus or location. Finally, as the day of the test increased, the probability of having shorter grunts increased (slope estimate ± se: −0.13 ± 0.04 and −0.04 ± 0.04, respectively, for day 2 and 3, Table 8), with no interaction with the type of stimulus. Concerning the acoustic spectral score (Figure 4E and Table 8): the probability of producing grunts with a lower acoustic spectral score depended of the type of stimulus and the spatial proximity, as the acoustic spectral score was more likely to decrease when approaching the object but not the human (Figure 4G).The higher the behavioral proximity score, the higher the probability of producing grunts with a lower acoustic spectral score, independently from the type of stimulus and location in the room (Figure 4H and Table 8). As the time during the test increased, the probability of producing grunts with a higher acoustic spectral score increased, independently from the type of stimulus or location (Figure 4I). Finally, as the day of the test increased, the probability of producing grunts with a lower acoustic spectral score increased independently of the type of stimulus (slope estimate ± se: −067 ± 0.26 and −0.51 ± 0.27, respectively, for day 2 and three, Table 8).

## Discussion

### No Evidence of a Preference Toward a Stimulus But Specific Human Directed Behaviors

In a V shaped arena Choice test, comparing the time spent close to and in contact with each stimulus (first behavioral response score, choicePC1), or the latency to reach the stimulus zone and exploring the stimulus zone (third behavioral response score, choicePC3), did not lead to significant differences between the types of stimuli. Neither was evidence for a consistent choice found when considering the first approach. Therefore, no consistent conclusion on a preference toward one of the stimuli can be drawn. Using the home pen to test for preference, as in mice for instance (37), may have led to different results, although the technical procedures would have been much more complicated. Indeed, male mice may show preferential attraction to different enrichment stimuli in such a situation (38). However, no particular negative behaviors associated with fear or stress were recorded during the test, thus the situation itself may not have been negative for our pigs. In addition, the absolute time spent close to each of the stimuli (between 73.9 and 100 s out of 300 s) or in contact with the stimulus (between 28.5 and 67.8 s out of 300 s, Supplementary Table 5) were high enough to conclude that the stimuli were attractive. In addition, two differences were apparent between the human and the object. The pigs more often entered the human zone than the object zone (second behavioral response score, choicePC2) and the number of times pigs laid down near the stimulus was human zone specific. Lying down is a sign of absence of stress in pigs (39), and the location was not by chance here. We may hypothesize that the human had reassuring effects, as has been found in studies on other farm animal species (19, 20). This would need to be confirmed, for example by using other non-invasive ways allowing positive emotional state to be monitored, such as heart rate and its variability. The novel structure of the testing pen compared to that experienced previously by the pigs (open pen) may have attracted their attention more than the familiar stimuli present in the pen.

### Behavioral Evidence for Positive Attractiveness of Both a Manipulable Object and a Familiar Human

Isolation/reunion tests allowed us to show differential responses according to the stimulus pigs were reunited with. Behavioral measures showed that both stimuli were attractive for the pigs, with a decrease in the time spent in the zone distant from the stimulus and an increase in the time spent in the stimulus zone when pigs were reunited with the human or the object compared to remaining alone in the experimental room. Furthermore, pigs remained immobile for a shorter time during the test and they had a lower locomotor activity when a stimulus was present. Remaining immobile (without exploring or watching a specific part of the room) may be associated to an attentive state or vigilance. Therefore, these changes in locomotor activity may be explained along with the time spent near the stimulus and are in line with the hypothesis of attraction to the stimuli. Beyond these general changes in behavior, pigs expressed discriminatory behaviors according to the stimulus present. Indeed, in response to a reunion with the human compared to the object, pigs were quicker to enter the stimulus zone, expressed a lower mobility and a higher exploration time. In response to a reunion with the object, pigs spent more time watching the exit door than exploring the room, a response equivalent to the reunion phase without any stimulus (i.e., isolation). Therefore, results suggest that the presence of the familiar human may prevent the pigs from expressing stress responses (more vigilance behavior and less exploration), a hypothesis strengthened by the fact that being reunited with the object or without any stimulus seem equivalent in terms of postural and locomotory behaviors.

### Acoustic Evidence of a High Arousal Positive Emotional State With the Human and a Low Arousal Negative Emotional State With the Object

We predicted that, if vocalizations reflect expression of the emotional state of the pigs, acoustic scores should be different when pigs are reunited with a stimulus compared to without one (isolated). In reaction to the reunion with the familiar human, the duration of grunts decreased and this was not the case with the object or when pigs remained alone. Shorter vocalizations have been associated with positive contexts compared to negative ones in many species (40), and especially shorter grunts in pigs (24, 28). We can compare the absolute values of grunt duration from the present study (250 ± 180 ms with human, 380 ± 220 ms with object, 330 ± 210 ms isolated, Supplementary Table 3) and other studies (negative vs. positive context (41): 480 ms vs. 280 ms; negative vs. positive context (24): ~430 ms vs. ~350 ms; anticipation of social reunions with pen mates (26): ~240 ms. Although comparisons must be made with caution, due to the material and methodological specificity of each study, the range of values we obtained with the human are in the range of other positively perceived situations. Behavior and acoustics together may allow us to conclude that being reunited with the human leads to a more positive context than reunion with the object. Since the human has previously been associated with positive tactile contacts, known to promote a positive state (14, 16), the presence of the human may engage the pigs in a positive anticipation of tactile interactions. Being reunited with the object appeared to lead to the expression of an emotional state not different from being without a stimulus (i.e., negative effect of isolation) even if it was attractive to some extent. Hence, behavioral and vocal clues do not seem to provide indications which point in the same direction. However, we can hypothesize that, even if the object is attractive, pigs may express frustration that they are not reunited with the human providing positive contacts. In addition, as shown by Villain et al. (26), even when two situations are behaviorally considered positive, pigs may rank the two situations and classify one of them as relatively negative. In fact, in Villain et al. (26), pigs vocally expressed the frustration of being reunited with the human and not their conspecifics. Here, a similar mechanism may be at play: pigs may express the frustration of being reunited with the inanimate object when they could have had positive contacts from a human instead.

During the reunion with either the object or the human, the spectral acoustic score of pig grunts decreased: grunts were composed of higher frequencies and a higher noise component, and this effect was greater with the human compared to the object. Changes in spectral components in response to changing contexts may be associated with the arousal of situations in mammals (26, 42). This may underline that the reunion with a stimulus promotes emotional states of high intensity in pigs, especially the reunion with a human. Villain et al. (26) showed that pigs were able to rapidly change the spectral properties of their grunts when anticipating positive events. The anticipation of a reunion with familiar conspecifics led to noisier grunts, whereas the anticipation of a reunion with a familiar human associated with positive contacts led to higher pitched grunts. In the present study, frequency and noise components of the grunt are closely intercorrelated, so it is not possible to discriminate between the two.

From the comparisons of grunt durations and spectral scores, we can summarize that being reunited with a familiar human at least alleviates the distress of isolation, but may also induce a high arousal and positive emotional state, through reassuring effects, while being reunited with a familiar object may induce a low arousal negative emotional state, after a social isolation. Thus, a positive relationship with a human seems to be more valuable as an enrichment for pigs. This may result from the relationship created through the numerous sessions of positive vocal and tactile interactions, as already shown in previous studies (16, 22). An inanimate object may not acquire similar properties. As a consequence, promoting social or pseudo-social enrichment in pigs is a good way to enhance their welfare.

### Experience and Spatial Location Predict Differences in Spectro-Temporal Features of Grunts Depending on the Stimulus

To investigate further, we studied which variables predicted vocal characteristics. From the model selection, we found that the type of stimulus (object or human) was among the best predictors of vocal expression (number of grunts, grunt rate, duration, and spectral score) and was the only consistent predictor explaining the temporal dynamic (grunt rate). Being reunited with a human (but not an object) is associated with more vocal production and at a higher rate. Morton (43) explained that the rhythm of a behavior can be positively linked to motivation of the producer, and thus a higher arousal. Villain et al. (26) showed that pigs had a higher grunt rate when anticipating the arrival of conspecifics, compared to a familiar human. In the present study, we would interpret the result in the direction of a higher motivation toward the human compared to the object.

Being reunited with the human and being close to them is likely to induce shorter grunts, whereas being reunited with the object and close to it is likely to induce a lower number of higher frequency and noisier grunts. This is in line with the more positive state, through reassuring effects, induced by the familiar human compared to the object after a short period of social isolation.

The behavioral proximity score, associated with the number of interactions and the time spent in contact with or near the stimulus, was a consistent predictor for both acoustic scores. Independently from the type of stimulus, the higher the behavioral proximity to the stimulus, the higher was the probability of producing shorter grunts with higher frequencies and noise components. This raises the possibility to monitor the degree of behavioral proximity to an enrichment by analyzing the structure of grunts (28).

The time during the test was also a predictor of the spectro-temporal features: the later in the test, the higher was the probability of producing longer, lower pitched and less noisy grunts (effect of interval index) and this result was independent from the type of stimulus. We can hypothesize that the positive effect of stimulus presence may be attenuated with time during the test and/or that negative effects of isolation from penmates may increase. In addition, since during the test the human did not interact with the pig as she would have done outside of the test situation, we can hypothesize either that the test makes the human more like an inanimate object and pigs may habituate to the stimulus, or that pigs may be frustrated if the human does not interact with them as in Tallet et al. (16). It would be interesting to investigate whether interacting with the pig during an Isolation/Reunion test may prolong the positive effect of the reunion with a familiar human after a 5 min isolation. Finally, over successive days grunts were more likely to be shorter, higher pitched and noisier, independently from the type of stimulus. This may have been linked to habituation to the test protocol.

### Is a Familiar Human More Than an Enrichment? Implications for Pig Welfare and Welfare Policies

Although paradigms generally used to quality an entity as “environmental enrichment” are usually performed in the home pen, our study shows similarities between what should be expected from an enrichment in the home pen and the responses we observed during the Isolation/Reunion test. Indeed, the reunion with a familiar object or human led to an attraction toward the stimulus and repeated contacts, as well as a decrease in attentive/vigilance behavior. These parameters are in line with the definition of what an enrichment should promote, that is to say a sustainable attraction and oral manipulations (5, 8). Therefore, it would be possible to extend the way we test for enrichment to other contexts than the home pen in future studies.

In addition, compared to an inanimate object, being with a human and/or close to the human provokes higher degrees of behavioral change in pigs (both spatial and vocal), and specific behavioral postures (lying down), associated with positive states. Regarding vocal behavior, although we showed that the behavioral proximity to the stimulus and vocal responses correlated, only the human presence led to positive shorter grunts during the reunion. Thus, analyzing vocal behavior enabled us to distinguish between the two kinds of stimuli and have insights into the emotional state of the pigs. This would imply that considering only postural/exploratory behaviors and describing attraction and contacts may not be enough to classify a stimulus as enrichment. We may need other non-invasive parameters such as vocalization monitoring, that do not entail a need to handle the animal.

With both postural/exploratory and vocal behaviors we conclude that, following a stressful isolation, only a familiar human and not an inanimate object is capable of generating a positive emotional state through reassuring properties. In a sense, human presence may be more than an enrichment and should be considered in further political decisions. Indeed, novelty is a paramount feature to promote a long term positive context and delay habituation effects (44). It is possible that interactions with the human may allow this feature, as a human is moving, talking and is unlikely to reproduce exactly the same gesture, at the same rhythm, which may contribute to promoting a higher level of stimulation than an object can provide. More studies are needed to better describe what are the most efficient human signals and behaviors that promote positive emotional states using a multimodal approach: voice? (45), shape? facial expression? [e.g. goats (46)], facial cues (47) or odors? [review in (48)], combinations of factors? (49).

## Conclusion

Using behavioral monitoring, this study showed that a manipulable inanimate object and a familiar human can be attractive for weaned pigs away from their rearing environment. Vocal monitoring showed that only the familiar human, but not the inanimate object, may alleviate the stress following of social isolation and induce a positive and high arousal emotional state when the pig is reunited with a familiar human, through reassuring effects. More studies should consider pseudo-social interactions between humans and pigs to enhance welfare, through a better relationship between the pigs and the humans. In order to be applicable on a larger scale, we must better understand the timing for the establishment of an effective human-pig relationship, as well as the most efficient signals triggering positive emotional states in pigs.

## Data Availability Statement

The datasets generated for this study can be found in online repositories. The names of the repository/repositories and accession number(s) can be found at: https://doi.org/10.15454/GDLDBH.

## Ethics Statement

The animal study was reviewed and approved by Ministry of Higher Education, Research and Innovation, APAFIS#17071-2018101016045373_V3.

## Author Contributions

AV, CT, and ML conceived and designed the experiment. AV, CT, and ML contributed to the writing of the manuscript. AV, CG, and ML: performed the experiment and collected and edited the acoustic and behavioral data. AV did the statistical analyses. All authors contributed to the article and approved the submitted version.

## Conflict of Interest

The authors declare that the research was conducted in the absence of any commercial or financial relationships that could be construed as a potential conflict of interest.
