# Supplementary material for: Manipulable Object and Human Contact: Preference and Modulation of Emotional States in Weaned Pigs
Source: Front Vet Sci. 2020 Nov 27;7:577433. doi: 10.3389/fvets.2020.577433 (PMC7728720; doi:10.3389/fvets.2020.577433)
Supplement: Supplementary file 1 [file Data_Sheet_1.PDF]

Supplementary Material

Manipulable object and human contact: preference and modulation of emotional states in weaned pigs

1 Supplementary tables

Supplementary table S1: Absolute values per group of parameters loaded to the Principal component analysis for the choice test. For each parameter, the number of observations (N) and the group –defined by a stimulus (Human or Object) during a day (Day 1 or Day2), is indicated. For each parameter, the mean, standard deviation (sd) and 95% confidence interval (ci) are indicated

			Number of visits in zone			Mean duration in zone (s)			Proportion of time in zone			Time spent in contact to the stimulus (s)		
Stim	Day of test	N	mean	sd	ci	mean	sd	ci	mean	sd	ci	mean	sd	ci
Human	Day 1	24	5.71	1.99	0.84	19.04	11.28	4.76	0.57	0.21	0.09	44.50	29.98	12.66
Human	Day 2	24	4.33	1.86	0.78	20.12	15.29	6.45	0.47	0.22	0.09	28.48	26.86	11.34
Object	Day 1	24	4.50	1.56	0.66	17.46	12.45	5.26	0.43	0.21	0.09	49.57	46.77	19.75
Object	Day 2	24	4.04	1.85	0.78	29.28	27.92	11.79	0.53	0.22	0.09	67.70	50.53	21.34

			Total time in zone (s)			Time spent exploring in zone (s)			Latency to approach zone (s)		
Stimulus	Day of test	N	mean	sd	ci	mean	sd	ci	mean	sd	ci
Human	Day 1	24	100.05	53.23	22.48	9.01	9.35	3.95	14.61	8.09	3.42
Human	Day 2	24	82.19	55.04	23.24	13.31	11.86	5.01	28.45	41.59	17.56
Object	Day 1	24	73.93	48.56	20.50	9.56	10.60	4.48	20.15	25.42	10.74
Object	Day 2	24	97.30	65.08	27.48	16.66	23.64	9.98	12.45	16.32	6.89

Supplementary table S2: Absolute values per group of parameters loaded to the Principal component analysis for the Isolation/Reunion test. For each parameter, the number of observations (N) and the group –defined according to a stimulus (Human or Object) and/or a day (Day 1 or Day2) and/or a phase of a test is indicated. For each parameter, the mean, standard deviation (sd) and 95% confidence interval (ci) are indicated.

			Time spent standing immobile (per min.)			Time spent looking at exit door (per min.)			Time spent in proximal zone (per min.)			Time spent in distal zone (per min.)		
Stimulus	Phase	N	mean	sd	ci	mean	sd	ci	mean	sd	ci	mean	sd	ci
Human	Isolation	24	2.480	0.750	0.317	1.374	0.654	0.276	1.612	0.921	0.389	2.323	0.912	0.385
Human	Reunion	24	1.254	1.075	0.454	1.310	1.251	0.528	3.429	0.435	0.184	1.125	1.074	0.454
No stimulus	Isolation	24	2.254	0.808	0.341	1.475	0.729	0.308	1.509	0.890	0.376	2.291	0.738	0.311
No stimulus	Reunion	24	2.114	0.882	0.373	3.059	1.036	0.437	3.122	0.553	0.233	1.614	1.013	0.428
Object	Isolation	24	2.073	0.738	0.312	1.252	0.585	0.247	1.861	0.633	0.267	2.451	0.655	0.277
Object	Reunion	24	2.067	0.937	0.396	2.837	1.219	0.515	1.371	1.004	0.424	1.808	1.000	0.422
Day of Test														
Day1		48	2.255	0.739	0.215	2.020	1.151	0.334	2.084	1.064	0.309	1.952	0.907	0.263
Day2		48	1.997	0.986	0.286	1.783	1.173	0.341	2.234	1.145	0.333	1.976	1.097	0.318
Day3		48	1.870	1.040	0.302	1.850	1.297	0.377	2.134	1.143	0.332	1.878	1.041	0.302

			Number of zone changes			Time spent exploring the room (per min.)			Latency to enter proximal zone		
Stimulus	Phase	N	mean	sd	mean		sd	ci	mean	sd	ci
Human	Isolation	24	2.079	0.476	0.201	2.831	0.502	0.212	229.568	103.919	43.881
Human	Reunion	24	1.941	0.451	0.190	2.410	0.569	0.240	195.342	279.449	118.001
No stimulus	Isolation	24	2.059	0.516	0.218	2.805	0.683	0.289	272.457	77.145	32.575
No stimulus	Reunion	24	1.937	0.389	0.164	2.267	0.651	0.275	248.653	273.280	115.396
Object	Isolation	24	2.227	0.318	0.134	3.195	0.520	0.220	255.447	91.274	38.542
Object	Reunion	24	1.923	0.632	0.267	2.677	0.791	0.334	555.383	117.831	49.756
Day of Test											
Day1		48	2.136	0.414	0.120	2.904	0.471	0.137	352.399	202.409	58.773
Day2		48	1.981	0.451	0.131	2.666	0.729	0.212	262.811	216.949	62.996
Day3		48	1.965	0.551	0.160	2.523	0.778	0.226	263.214	210.648	61.166

Supplementary table S3: Acoustic values for acoustic scores per significantly different groups (Stimulus, phase of test and location). The number of vocalisations per group is indicated and mean, standard deviation (sd), and 95% confidence interval (ci) are indicated for the vocalisation duration and all the spectral parameters used to build the spectral acoustic score (PC1)

			Mean (Hz)			Centroid (Hz)			Mean Dominant Frequency (KHz)		
Stimulus	Phase of Test	N	mean	sd	ci	mean	sd	ci	mean	sd	ci
Human	Isolation	673	975.576	240.935	18.236	975.576	240.935	18.236	0.295	0.026	0.002
Human	Reunion	1302	1135.032	357.416	19.432	1135.032	357.416	19.432	0.307	0.065	0.004
No stimulus	Isolation	775	1002.804	280.949	19.811	1002.804	280.949	19.811	0.295	0.037	0.003
No stimulus	Reunion	1286	1018.402	307.527	16.824	1018.402	307.527	16.824	0.301	0.039	0.002
Object	Isolation	755	973.887	294.108	21.013	973.887	294.108	21.013	0.293	0.040	0.003
Object	Reunion	975	1042.705	292.903	18.408	1042.705	292.903	18.408	0.299	0.040	0.003
Location	Stimulus	N									
Away	Human	537	1157.254	382.488	32.424	1157.254	382.488	32.424	0.308	0.059	0.005
Away	Object	415	1034.390	303.160	29.253	1034.390	303.160	29.253	0.304	0.040	0.004
Close	Human	604	1127.452	337.411	26.963	1127.452	337.411	26.963	0.304	0.037	0.003
Close	Object	305	1074.428	257.501	29.014	1074.428	257.501	29.014	0.293	0.036	0.004
			Inter Quartile Range (Hz)			spectrum standard deviation			Call duration (s)		
Stimulus	Phase of Test	N	mean	sd	ci	mean	sd	ci	mean	sd	ci
Human	Isolation	673	636.416	504.019	38.148	1473.751	254.950	111.544	0.379	0.222	0.017
Human	Reunion	1302	941.105	768.408	41.777	1603.690	296.579	87.190	0.251	0.185	0.010
No stimulus	Isolation	775	719.745	617.101	43.514	1490.436	274.302	105.097	0.362	0.219	0.015
No stimulus	Reunion	1286	749.850	675.886	36.975	1493.353	281.679	81.696	0.333	0.207	0.011
Object	Isolation	755	650.317	623.269	44.530	1461.763	274.854	104.436	0.385	0.241	0.017
Object	Reunion	975	776.602	633.862	39.836	1524.787	266.550	95.829	0.385	0.222	0.014
Location	Stimulus	N									
Away	Human	537	996.992	840.728	71.269	1617.188	313.709	137.089	0.288	0.196	0.017
Away	Object	415	755.197	653.837	63.091	1512.223	265.977	145.919	0.380	0.222	0.021
Close	Human	604	921.476	723.267	57.796	1600.669	287.511	127.910	0.200	0.145	0.012
Close	Object	305	835.885	560.337	63.136	1560.115	234.007	175.787	0.366	0.206	0.023
			Shannon entropy			Spectral Flatness			Entropy		
Stimulus	Phase of Test	N	mean	sd	ci	mean	sd	ci	mean	sd	ci

Human	Isolation	673	0.651	0.067	0.005	0.270	0.087	0.007	0.501	0.049	0.004
Human	Reunion	1302	0.686	0.079	0.004	0.319	0.114	0.006	0.517	0.058	0.003
No stimulus	Isolation	775	0.655	0.072	0.005	0.277	0.098	0.007	0.504	0.053	0.004
No stimulus	Reunion	1286	0.660	0.077	0.004	0.280	0.104	0.006	0.506	0.058	0.003
Object	Isolation	755	0.647	0.074	0.005	0.267	0.100	0.007	0.498	0.054	0.004
Object	Reunion	975	0.669	0.074	0.005	0.291	0.098	0.006	0.516	0.054	0.003
Location	Stimulus	N									
Away	Human	537	0.686	0.085	0.007	0.322	0.119	0.010	0.521	0.062	0.005
Away	Object	415	0.665	0.072	0.007	0.285	0.098	0.009	0.512	0.053	0.005
Close	Human	604	0.689	0.076	0.006	0.320	0.112	0.009	0.515	0.056	0.004
Close	Object	305	0.685	0.068	0.008	0.306	0.089	0.010	0.527	0.049	0.005

Supplementary table S4: After model validation, effects of explanatory variables were computed using the ‘Anova’ function (‘car’ R package), running Type II Wald chisquare trials. All linear models were computed using the ’lmer’ function, taking into account repeated observations as random factors (individual). One model had a binary response variable and one model was on counting was computed with a generalized model taking into account repeated observations as random factors, respectively using a Binomial and a Poisson distribution (see models 2 and 4 when indicated). P values were considered significant when below 0.05.

	Chisq	Df	Pr.Chisq.
**Model 1: PC1 of choice test**			
Stimulus	0.284	1	0.594
Day	0.086	1	0.769
Position of human (left vs. right)	0.073	1	0.787
Stimulus: Day	6.300	1	0.012
**Model 1: PC2 of choice test**			
Stimulus	7.286	1	0.007
Day	3.252	1	0.071
Position of human (left vs. right)	0.166	1	0.683
Stimulus: Day	0.715	1	0.398
**Model 1: PC3 of choice test**			
Stimulus	1.512	1	0.219
Day	0.567	1	0.451
Position of human (left vs. right)	0.019	1	0.891
Stimulus: Day	1.973	1	0.160
**Model 2 : first approach (binomial)**			
Day	3.440	1	0.064
Position of human (left vs. right)	1.831	1	0.176
**Model 3 : Isolation/Reunion test - behaviour PC1**			
Stimulus	24.063	2	<0.001
Phase of Test	33.834	1	<0.001
Day	10.071	2	0.007
Stimulus : Phase of Test	16.565	2	<0.001
Stimulus : Day	3.035	4	0.552
Phase of Test : Day	1.474	2	0.479
**Model 3 : Isolation/Reunion test - behaviour PC2**			
Stimulus	16.576	2	<0.001
Phase of Test	45.999	1	<0.001
Day	0.994	2	0.608
Stimulus : Phase of Test	41.531	2	<0.001
Stimulus : Day	4.211	4	0.378
Phase of Test : Day	4.887	2	0.087
**Model 3 : Isolation/Reunion test - behaviour PC3**			
Stimulus	29.694	2	<0.001
Phase of Test	44.445	1	<0.001
Day	0.201	2	0.904
Stimulus : Phase of Test	36.383	2	<0.001
Stimulus : Day	7.294	4	0.121
Phase of Test : Day	2.922	2	0.232
**Model 3 : Acoustic Spectral Score (PC1)**			
Stimulus	46.813	2	<0.001
Phase of Test	69.814	1	<0.001
Day	12.796	2	0.002
Stimulus : Phase of Test	45.131	2	<0.001

Stimulus : Day	26.773	4	<0.001
Phase of Test : Day	1.301	2	0.522
**Model 3 : Acoustic grunt duration (log)**			
Stimulus	257.550	2	<0.001
Phase of Test	129.889	1	<0.001
Day	19.919	2	<0.001
Stimulus : Phase of Test	210.139	2	<0.001
Stimulus : Day	4.579	4	0.333
Phase of Test : Day	4.761	2	0.093

Supplementary table S5: Post hoc tests on models following significant interactions or single effects of explanatory variables. Each model is indicated, all post hoc tests were computed using the ’lsmeans’ function with Tukey correction for multiple testing. P values were considered significant when below 0.05.

Contrast	Estimate	SE	DF	t.ratio	p.value
**Model 1: PC1 of choice test**					
Human, day1 - Object, day1	-0.716	0.512	68	-1.398	0.505
Human, day1 - Human, day2	-0.803	0.513	68	-1.563	0.406
Human, day1 - Object, day2	0.300	0.513	68	0.584	0.937
Object, day1 - Human, day2	-0.086	0.513	68	-0.168	0.998
Object, day1 - Object, day2	1.016	0.513	68	1.980	0.206
Human, day2 - Object, day2	1.102	0.512	68	2.151	0.148
**Model 1: PC2 of choice test**					
Human - Object	0.475	0.176	68	2.699	0.009
Day1 - Day2	0.318	0.177	68	1.803	0.076
**Model 3 : Isolation/Reunion test - behaviour PC1**					
Human,Isolation - Object,Isolation	-0.041	0.330	109	-0.125	1.000
Human,Isolation - No stimulus,Isolation	0.192	0.330	109	0.582	0.992
Human,Isolation - Human,Reunion	-2.088	0.330	109	-6.332	<0.001
Human,Isolation - Object,Reunion	-1.082	0.330	109	-3.282	<0.001
Human,Isolation - No stimulus,Reunion	-0.001	0.330	109	-0.004	1.000
Object,Isolation - No stimulus,Isolation	0.233	0.330	109	0.708	0.981
Object,Isolation - Human,Reunion	-2.047	0.330	109	-6.207	<0.001
Object,Isolation - Object,Reunion	-1.041	0.330	109	-3.156	0.025
Object,Isolation - No stimulus,Reunion	0.040	0.330	109	0.121	1.000
No stimulus,Isolation - Human,Reunion	-2.280	0.330	109	-6.914	<0.001
No stimulus,Isolation - Object,Reunion	-1.274	0.330	109	-3.864	0.003
No stimulus,Isolation - No stimulus,Reunion	-0.193	0.330	109	-0.587	0.992
Human,Reunion - Object,Reunion	1.006	0.330	109	3.050	0.033
Human,Reunion - No stimulus,Reunion	2.087	0.330	109	6.328	<0.001
Object,Reunion - No stimulus,Reunion	1.081	0.330	109	3.277	0.017
**Model 3 : Isolation/Reunion test - behaviour PC1**					
Day1 - Day2	-0.552	0.233	109	-2.366	0.051
Day1 - Day3	-0.703	0.233	109	-3.015	0.009
Day2 - Day3	-0.151	0.233	109	-0.648	0.794
**Model 3 : Isolation/Reunion test - behaviour PC2**					
Human,Isolation - Object,Isolation	0.115	0.258	109	0.443	0.998

Human,Isolation - No stimulus,Isolation	-0.586	0.258	109	-2.269	0.216
Human,Isolation - Human,Reunion	-0.154	0.258	109	-0.598	0.991
Human,Isolation - Object,Reunion	1.104	0.258	109	4.273	0.001
Human,Isolation - No stimulus,Reunion	1.614	0.258	109	6.246	<0.001
Object,Isolation - No stimulus,Isolation	-0.701	0.258	109	-2.712	0.081
Object,Isolation - Human,Reunion	-0.269	0.258	109	-1.041	0.903
Object,Isolation - Object,Reunion	0.990	0.258	109	3.830	0.003
Object,Isolation - No stimulus,Reunion	1.500	0.258	109	5.803	<0.001
No stimulus,Isolation - Human,Reunion	0.432	0.258	109	1.671	0.554
No stimulus,Isolation - Object,Reunion	1.691	0.258	109	6.542	<0.001
No stimulus,Isolation - No stimulus,Reunion	2.200	0.258	109	8.515	<0.001
Human,Reunion - Object,Reunion	1.259	0.258	109	4.871	<0.001
Human,Reunion - No stimulus,Reunion	1.769	0.258	109	6.844	<0.001
Object,Reunion - No stimulus,Reunion	0.510	0.258	109	1.973	0.365
**Model 3 : Isolation/Reunion test - behaviour PC3**					
Human,Isolation - Object,Isolation	-0.150	0.210	109	-0.713	0.980
Human,Isolation - No stimulus,Isolation	-0.032	0.210	109	-0.153	1.000
Human,Isolation - Human,Reunion	1.002	0.210	109	4.760	<0.001
Human,Isolation - Object,Reunion	1.446	0.210	109	6.872	<0.001
Human,Isolation - No stimulus,Reunion	-0.200	0.210	109	-0.951	0.932
Object,Isolation - No stimulus,Isolation	0.118	0.210	109	0.560	0.993
Object,Isolation - Human,Reunion	1.152	0.210	109	5.473	<0.001
Object,Isolation - Object,Reunion	1.596	0.210	109	7.585	<0.001
Object,Isolation - No stimulus,Reunion	-0.050	0.210	109	-0.238	1.000
No stimulus,Isolation - Human,Reunion	1.034	0.210	109	4.913	<0.001
No stimulus,Isolation - Object,Reunion	1.478	0.210	109	7.025	<0.001
No stimulus,Isolation - No stimulus,Reunion	-0.168	0.210	109	-0.798	0.967
Human,Reunion - Object,Reunion	0.444	0.210	109	2.112	0.289
Human,Reunion - No stimulus,Reunion	-1.202	0.210	109	-5.711	<0.001
Object,Reunion - No stimulus,Reunion	-1.646	0.210	109	-7.823	<0.001
**Model 3 : Acoustic Spectral Score (PC1)**					
Human,isolation - No stimulus,isolation	0.177	0.129	5735	1.377	0.741
Human,isolation - Object,isolation	-0.056	0.131	5737	-0.427	0.998
No stimulus,isolation - Object,isolation	-0.233	0.126	5736	-1.854	0.431
Human,isolation - Human,reunion	1.088	0.117	5743	9.289	<0.001
Human,isolation - No stimulus,reunion	0.182	0.117	5737	1.550	0.632
Human,isolation - Object,reunion	0.574	0.123	5737	4.660	<0.001
No stimulus,isolation - Human,reunion	0.911	0.112	5741	8.163	<0.001
No stimulus,isolation - No stimulus,reunion	0.004	0.112	5738	0.038	1.000
No stimulus,isolation - Object,reunion	0.396	0.118	5741	3.346	0.011
Object,isolation - Human,reunion	1.144	0.114	5746	10.012	<0.001
Object,isolation - No stimulus,reunion	0.238	0.113	5739	2.096	0.289
Object,isolation - Object,reunion	0.629	0.120	5740	5.251	<0.001
Human,reunion - No stimulus,reunion	-0.907	0.098	5747	-9.223	<0.001
Human,reunion - Object,reunion	-0.515	0.105	5745	-4.880	<0.001

No stimulus,reunion - Object,reunion	0.392	0.105	5744	3.725	0.003
**Model 3 : Acoustic Spectral Score (PC1)**					
Human, Day1 - No stimulus, Day1	-0.025	0.464	24	-0.055	1.000
Human, Day1 - Object, Day1	-0.055	0.464	24	-0.119	1.000
Human, Day1 - Human, Day2	0.631	0.463	23	1.364	0.900
Human, Day1 - No stimulus, Day2	0.117	0.136	5736	0.856	0.995
Human, Day1 - Object, Day2	-0.139	0.468	24	-0.298	1.000
Human, Day1 - Human, Day3	0.361	0.466	24	0.775	0.997
Human, Day1 - No stimulus, Day3	-0.193	0.465	24	-0.414	1.000
Human, Day1 - Object, Day3	0.331	0.147	5748	2.259	0.368
No stimulus, Day1 - Object, Day1	-0.030	0.465	24	-0.064	1.000
No stimulus, Day1 - Human, Day2	0.656	0.464	24	1.414	0.881
No stimulus, Day1 - No stimulus, Day2	0.142	0.462	23	0.307	1.000
No stimulus, Day1 - Object, Day2	-0.114	0.151	5734	-0.755	0.998
No stimulus, Day1 - Human, Day3	0.387	0.146	5741	2.656	0.164
No stimulus, Day1 - No stimulus, Day3	-0.167	0.466	24	-0.359	1.000
No stimulus, Day1 - Object, Day3	0.356	0.465	24	0.766	0.997
Object, Day1 - Human, Day2	0.686	0.132	5738	5.181	0.000
Object, Day1 - No stimulus, Day2	0.172	0.461	23	0.373	1.000
Object, Day1 - Object, Day2	-0.084	0.467	24	-0.180	1.000
Object, Day1 - Human, Day3	0.416	0.467	24	0.892	0.991
Object, Day1 - No stimulus, Day3	-0.137	0.138	5743	-0.996	0.986
Object, Day1 - Object, Day3	0.386	0.464	24	0.832	0.995
Human, Day2 - No stimulus, Day2	-0.514	0.460	23	-1.119	0.965
Human, Day2 - Object, Day2	-0.770	0.466	24	-1.651	0.768
Human, Day2 - Human, Day3	-0.270	0.466	24	-0.579	1.000
Human, Day2 - No stimulus, Day3	-0.824	0.133	5742	-6.171	0.000
Human, Day2 - Object, Day3	-0.300	0.463	24	-0.647	0.999
No stimulus, Day2 - Object, Day2	-0.256	0.465	24	-0.551	1.000
No stimulus, Day2 - Human, Day3	0.245	0.464	24	0.527	1.000
No stimulus, Day2 - No stimulus, Day3	-0.309	0.462	23	-0.669	0.999
No stimulus, Day2 - Object, Day3	0.214	0.135	5744	1.584	0.814
Object, Day2 - Human, Day3	0.501	0.154	5738	3.253	0.032
Object, Day2 - No stimulus, Day3	-0.053	0.469	25	-0.114	1.000
Object, Day2 - Object, Day3	0.470	0.468	25	1.004	0.982
Human, Day3 - No stimulus, Day3	-0.554	0.468	24	-1.184	0.953
Human, Day3 - Object, Day3	-0.030	0.467	24	-0.065	1.000
No stimulus, Day3 - Object, Day3	0.524	0.466	24	1.125	0.964
**Model 3 : Acoustic grunt duration (log)**					
Human,isolation - No stimulus,isolation	0.080	0.031	5734	2.604	0.096
Human,isolation - Object,isolation	0.029	0.031	5736	0.943	0.935
No stimulus,isolation - Object,isolation	-0.051	0.030	5735	-1.685	0.542
Human,isolation - Human,reunion	0.513	0.028	5741	18.340	<0.001
Human,isolation - No stimulus,reunion	0.122	0.028	5735	4.353	<0.001
Human,isolation - Object,reunion	0.023	0.029	5736	0.795	0.969
No stimulus,isolation - Human,reunion	0.433	0.027	5739	16.250	<0.001
No stimulus,isolation - No stimulus,reunion	0.042	0.027	5736	1.560	0.625
No stimulus,isolation - Object,reunion	-0.057	0.028	5739	-2.008	0.338
Object,isolation - Human,reunion	0.483	0.027	5744	17.721	<0.001
Object,isolation - No stimulus,reunion	0.092	0.027	5737	3.413	0.008
Object,isolation - Object,reunion	-0.006	0.029	5738	-0.214	1.000

Human,reunion - No stimulus,reunion	-0.391	0.023	5745	-16.665	<0.001
Human,reunion - Object,reunion	-0.489	0.025	5743	-19.448	<0.001
No stimulus,reunion - Object,reunion	-0.098	0.025	5742	-3.921	0.001
**Model 3 : Acoustic grunt duration (log)**					
Day1 - Day2	0.076	0.020	5734	3.892	<0.001
Day1 - Day3	0.052	0.020	5742	2.604	0.025
Day2 - Day3	-0.024	0.020	5739	-1.240	0.430

Supplementary table S6: Table of model estimates, following significant effect of explanatory variables. All estimates were calculated using the ‘lsmeans’ function (‘lmerTest’ R package) and are presented per relevant group lsmean, SE, DF, lower.CI upper.CI respectively represent mean estimates, standard error, degrees of freedom and lower and upper limit of 95% confidence interval

Factor 1	Factor 2	Lsmean	SE	DF	Lower.CL	Upper.CL
**Model 1: PC1 of choice test**						
Human	Day1	-0.305	0.363	90.995	-1.025	0.416
Object	Day1	0.412	0.363	90.995	-0.309	1.132
Human	Day2	0.498	0.363	90.995	-0.223	1.218
Object	Day2	-0.605	0.363	90.995	-1.325	0.116
**Model 1: PC2 of choice test**						
Human	-	0.238	0.200	34.646	-0.169	0.644
Object	-	-0.238	0.200	34.646	-0.644	0.169
-	Day1	0.159	0.200	34.731	-0.248	0.566
-	Day2	-0.159	0.200	34.731	-0.566	0.248
**Model 2 : first approach (binomial)**						
Day1	-	-0.211	0.421	Inf	-1.036	0.613
Day2	-	0.961	0.465	Inf	0.051	1.872
**Model 3 : Isolation/Reunion test - behaviour PC1**						
Human	Isolation	-0.406	0.291	117.573	-0.982	0.170
Object	Isolation	-0.329	0.291	117.573	-0.905	0.247
No stimulus	Isolation	-0.686	0.291	117.573	-1.262	-0.110
Human	Reunion	1.277	0.291	117.573	0.701	1.853
Object	Reunion	0.271	0.291	117.573	-0.305	0.848
No stimulus	Reunion	-0.128	0.291	117.573	-0.704	0.448
**Model 3 : Isolation/Reunion test - behaviour PC1**						
Day1	-	-0.473	0.219	71.500	-0.910	-0.036
Day2	-	0.124	0.219	71.500	-0.313	0.562
Day3	-	0.349	0.219	71.500	-0.089	0.786
**Model 3 : Isolation/Reunion test - behaviour PC2**						
Human	Isolation	0.031	0.202	121.263	-0.370	0.432
Object	Isolation	-0.154	0.202	121.263	-0.555	0.247
No stimulus	Isolation	0.490	0.202	121.263	0.089	0.891
Human	Reunion	1.095	0.202	121.263	0.694	1.496
Object	Reunion	-0.047	0.202	121.263	-0.448	0.354
No stimulus	Reunion	-1.415	0.202	121.263	-1.816	-1.014
**Model 3 : Isolation/Reunion test - behaviour PC3**						
Human	Isolation	-0.496	0.177	91.865	-0.848	-0.144
Object	Isolation	-0.543	0.177	91.865	-0.895	-0.191
No stimulus	Isolation	-0.605	0.177	91.865	-0.957	-0.253
Human	Reunion	0.390	0.177	91.865	0.038	0.742
Object	Reunion	1.262	0.177	91.865	0.910	1.614
No stimulus	Reunion	-0.009	0.177	91.865	-0.361	0.343
**Model 3 : Acoustic Spectral Score (PC1)**						
Human	isolation	0.319	0.204	31.821	-0.096	0.735

No stimulus	isolation	0.142	0.201	30.212	-0.269	0.552
Object	isolation	0.375	0.202	30.677	-0.037	0.787
Human	reunion	-0.769	0.194	26.023	-1.164	-0.374
No stimulus	reunion	0.138	0.194	25.830	-0.257	0.532
Object	reunion	-0.254	0.197	27.835	-0.656	0.148
**Model 3 : Acoustic Spectral Score (PC1)**						
Human	Day1	0.106	0.328	23.664	-0.572	0.783
No stimulus	Day1	0.131	0.330	24.068	-0.549	0.812
Object	Day1	0.161	0.328	23.596	-0.516	0.838
Human	Day2	-0.525	0.327	23.254	-1.200	0.150
No stimulus	Day2	-0.011	0.324	22.550	-0.680	0.659
Object	Day2	0.245	0.333	25.181	-0.443	0.933
Human	Day3	-0.255	0.332	24.806	-0.941	0.431
No stimulus	Day3	0.299	0.330	24.084	-0.382	0.980
Object	Day3	-0.225	0.329	23.838	-0.904	0.454
**Model 3 : Acoustic grunt duration (log)**						
Human	isolation	-1.153	0.054	29.113	-1.263	-1.042
No stimulus	isolation	-1.233	0.053	27.912	-1.342	-1.124
Object	isolation	-1.182	0.054	28.262	-1.292	-1.073
Human	reunion	-1.665	0.052	24.753	-1.771	-1.559
No stimulus	reunion	-1.274	0.052	24.605	-1.380	-1.169
Object	reunion	-1.176	0.052	26.121	-1.283	-1.069
**Model 3 : Acoustic grunt duration (log)**						
Day1	-	-1.238	0.051	23.156	-1.343	-1.133
Day2	-	-1.314	0.051	23.035	-1.419	-1.209
Day3	-	-1.290	0.051	23.406	-1.395	-1.184

Supplementary table S7: Model selection table. Only the equivalent best models after selections are shown (i.e Delta AICc<2) as well as the null model. All models are ranked. Estimates of each covariate when present in a given model are given, a '+' means the term is present in the model, DF, LogLik, AICc, Delta AICc and weight of models. 'Stim', 'Loc.' and 'Behav. Prox.' refer to 'Stimulus', 'Location' and 'behavioural proximity'. When the null model is in the best selected model, then we cannot extract any predictor.

Response variable	rank	Intercept	Stim	Day	Loc.	Int. Index	- Behav. Prox.	Stim :Day	Stim :Loc.	Stim :Int. Index	Stim : - Behav. Prox.	Loc : - Behav. Prox.	df	logLik	AICc	delta	weight
**Total Number grunts (Poisson)**	1	2.77	+	+	+		0.03		+		+		9	-487.236	994.751	0.000	0.426
	2	2.77	+	+	+		0.07		+		+	+	10	-486.094	995.008	0.257	0.375
	66	2.73	NULL										2	-581.376	1166.891	172.140	0.000
**Grunt rate (log)**	1	-1.56	+										4	-176.946	362.117	0.000	0.193
	2	-1.56	+				0.04						5	-176.716	363.774	1.656	0.084
	119	-1.77	NULL										3	-187.641	381.416	19.299	0.000
**Grunt duration (log)**	1	-1.50	+	+	+	0.11	-0.05		+	+			11	-1600.120	3222.382	0.000	0.172
	2	-1.49	+	+	+	0.10	-0.05		+				10	-1601.159	3222.436	0.054	0.167
	3	-1.50	+	+	+	0.12	-0.06		+	+		+	12	-1599.841	3223.851	1.470	0.082
	4	-1.50	+	+	+	0.11	-0.06		+	+	+		12	-1599.848	3223.866	1.484	0.082
	5	-1.49	+	+	+	0.10	-0.06		+				11	-1600.872	3223.887	1.505	0.081
	6	-1.49	+	+	+	0.10	-0.06		+		+		11	-1600.948	3224.039	1.657	0.075
	137	-1.49	NULL										3	-1814.290	3634.593	412.211	0.000

continue…

Response variable	rank	Intercept	Stim	Day	Loc.	Int. Index	-Behav. Prox. 1	Stim :Day	Stim :Loc.	Stim :Int. Index	Stim : -Behav. Prox. 1	Loc : -Behav. Prox. 1	df	logLik	AICc	delta	weight
**Acoustic spectral score (PC1)**	1	-0.18	+	+	+	0.27	-0.28		+				10	-4318.392	8656.904	0.000	0.101
	2	-0.15	+	+	+	0.28	-0.16		+		+		11	-4317.445	8657.032	0.128	0.095
	3	-0.31	+	+	+	0.28	-0.28	+	+				12	-4316.490	8657.149	0.245	0.089
	4	-0.30	+	+	+	0.28	-0.17	+	+		+		13	-4315.669	8657.534	0.630	0.074
	5	-0.20	+	+	+	0.31	-0.28		+	+			11	-4318.078	8658.298	1.394	0.050
	6	-0.17	+	+	+	0.30	-0.17		+	+	+		12	-4317.192	8658.553	1.649	0.044
	7	-0.33	+	+	+	0.31	-0.28	+	+	+			13	-4316.190	8658.578	1.674	0.044
	8	-0.15	+	+	+	0.27	-0.13		+		+	+	12	-4317.346	8658.860	1.956	0.038
	9	-0.19	+	+	+	0.27	-0.27		+				11	-4318.362	8658.866	1.962	0.038
	137	-0.38	NULL										3	-4346.453	8698.918	42.014	0.000
**More than 1 grunt per interval *(Binomial)***	1	1.49	+			-0.21							4	-153.427	314.996	0.000	0.071
	2	1.46	+										3	-154.502	315.090	0.094	0.068
	3	1.77	+	+									5	-153.031	316.277	1.282	0.038
	4	1.63	+		+	-0.22							5	-153.131	316.476	1.480	0.034
	5	1.79	+	+		-0.20							6	-152.124	316.550	1.554	0.033
	6	1.56	+		+								4	-154.291	316.725	1.730	0.030
	7	1.19	NULL										2	-156.355	316.752	1.756	0.030
	8	1.50	+			-0.22	0.08						5	-153.319	316.853	1.857	0.028
	9	1.46	+				0.08						4	-154.410	316.963	1.967	0.027

39

2 Supplementary figures

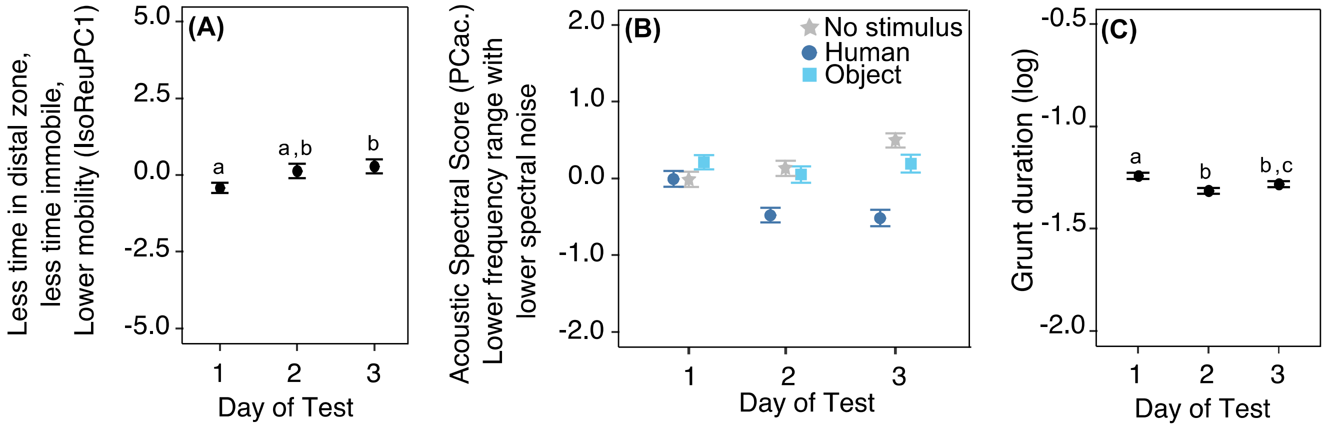

Supplementary Figure S1: Effect of the day of the test on vocal and spatial behavior of piglets during isolation/reunion tests. Mean +- se of the first behavioral score (A), the acoustic spectral score of grunts (B) and grunt duration (C), according to the day and/or the type of reunion (B, dark blue circles for human, light blue square for object and grey stars for no stimulus). Different letters show significantly different groups ($p < 0.05$). All model estimates, anova tables and results of post hoc tests are available in supplementary tables S4-S6.
